# Hemoglobin oxidation generates globin-derived peptides in atherosclerotic lesions and intraventricular hemorrhage of the brain, provoking endothelial dysfunction

**DOI:** 10.1038/s41374-020-0403-x

**Published:** 2020-02-13

**Authors:** Niké Posta, Éva Csősz, Melinda Oros, Dávid Pethő, László Potor, Gergő Kalló, Zoltán Hendrik, Katalin Éva Sikura, Gábor Méhes, Csaba Tóth, József Posta, György Balla, József Balla

**Affiliations:** 1 Department of Internal Medicine, University of Debrecen, Debrecen, 4032 Hungary; 2 Department of Cardiology and Cardiac Surgery, University of Debrecen, Debrecen, 4032 Hungary; 3 University of Debrecen, Kálmán Laki Doctoral School of Biomedical and Clinical Sciences, Debrecen, Hungary; 4 Department of Biochemistry and Molecular Biology, Faculty of Medicine, University of Debrecen, Debrecen, 4032 Hungary; 5 HAS-UD Vascular Biology and Myocardial Pathophysiology Research Group, Hungarian Academy of Sciences, Debrecen, Hungary; 6 Department of Pediatrics, University of Debrecen, Debrecen, 4032 Hungary; 7 Department of Pathology, University of Debrecen, Debrecen, 4032 Hungary; 8 Department of Vascular Surgery, University of Debrecen, Debrecen, 4032 Hungary; 9 Department of Inorganic and Analytical Chemistry, University of Debrecen, Debrecen, 4032 Hungary

**Keywords:** Vascular diseases, Atherosclerosis

## Abstract

The lysis of red blood cells was shown to occur in human ruptured atherosclerotic lesions and intraventricular hemorrhage (IVH) of the brain. Liberated cell-free hemoglobin was found to undergo oxidation in both pathologies. We hypothesize that hemoglobin-derived peptides are generated during hemoglobin oxidation both in complicated atherosclerotic lesions and IVH of the brain, triggering endothelial cell dysfunction. Oxidized hemoglobin and its products were followed with spectrophotometry, LC–MS/MS analysis and detection of the cross-linking of globin chains in complicated atherosclerotic lesions of the human carotid artery and the hemorrhaged cerebrospinal liquid of preterm infants. The vascular pathophysiologic role of oxidized hemoglobin and the resultant peptides was assessed by measuring endothelial integrity, the activation of endothelial cells and the induction of proinflammatory genes. Peptide fragments of hemoglobin (VNVDEVGGEALGRLLVVYPWTQR, LLVVYPWTQR, MFLSFPTTK, VGAHAGEYGAELERMFLSFPTTK, and FLASVSTVLTSKYR) were identified in ruptured atherosclerotic lesions and in IVH of the human brain. Fragments resulting from the oxidation of hemoglobin were accompanied by the accumulation of ferryl hemoglobin. Similar to complicated atherosclerotic lesions of the human carotid artery, a high level of oxidized and cross-linked hemoglobin was observed in the cerebrospinal fluid after IVH. Haptoglobin inhibited hemoglobin fragmentation provoked by peroxide. The resultant peptides failed to bind haptoglobin or albumin. Peptides derived from hemoglobin oxidation and ferryl hemoglobin induced intercellular gap formation, decreased junctional resistance in the endothelium, and enhanced monocyte adhesion to endothelial cells. Enhanced expression of TNF and the activation of NLRP3 and CASP1 followed by the increased generation of IL-1β and nuclear translocation of the NF-κβ transcription factor occurred in response to hemoglobin-derived peptides, and ferryl hemoglobin in endothelium was upregulated in both pathologies. We conclude that the oxidation of hemoglobin in complicated atherosclerotic lesions and intraventricular hemorrhage of the brain generates peptide fragments and ferryl hemoglobin with the potential to trigger endothelial cell dysfunction.

## Abstract (web summary)

Generation of peptide fragments by oxidation of hemoglobin to ferryl hemoglobin occurs in ruptured atherosclerotic lesion in human and in intraventricular hemorrhage of brain in preterm infants. Peptide fragments of hemoglobin provoke intercellular gap formation and enhance monocyte adhesion to endothelial cell. Activation of endothelium via NF-κβ leads to TNF and IL-1β production, induction of NLRP3 and CASP1.

## Introduction

The lysis of red blood cells occurs in several pathologic conditions [1]. When hemolysis occurs, cell-free hemoglobin α_2_β_2_ tetramers dissociate into α, β dimers, which are oxidized into methemoglobin (Fe^3+^) [2, 3]. Exposure of extracellular hemoglobin to reactive oxygen species, e.g., hydrogen peroxide (H_2_O_2_), or lipid hydroperoxide can lead to the formation of short-lived ferryl (Fe^4+^ = O^2−^) hemoglobin [4, 5]. Ferryl hemoglobin has the propensity to form free radicals in the alpha and beta chain of the globin [6–8]. Finally, globin–globin cross-links are formed by globin-centered radicals, resulting in hemoglobin dimers, tetramers and multimers, and heme (Fe^2+^). In this study, we refer to these globin-modified molecules as ferryl hemoglobin.

Plaque materials from atheroma with oxidative properties lead to the lysis of red blood cells, liberating hemoglobin in ruptured lesions [9]. Intraplaque hemorrhage was shown to result from the rupture of immature neovessels developing from the vasa vasorum [10–12]. Recently, Delbosc et al. showed that in early-stage atheroma, intimal red blood cell infiltration has an initial role in triggering foam cell formation and intimal oxidation [13]. Our research group previously described the accumulation of oxidized hemoglobin (ferryl hemoglobin) in the atherosclerotic lesion [9] and demonstrated that ferryl hemoglobin has the potential to activate the inflammatory response in the resident cells of the arterial wall [14]. This activation leads to increased endothelial cell permeability and enhanced monocyte adhesion [14, 15].

Heme oxygenase 1 is a key antioxidant enzyme that exhibits protective effects in endothelial cells [2, 16–19] upon exposure to heme or ferryl hemoglobin [2, 15, 20]. Heme oxygenase 1 was found to accumulate in human atherosclerotic lesions [21]. Its cytoprotective propensities make heme oxygenase 1 a potential target in the treatment of cardiovascular diseases [22–24].

Hemoglobin was shown to be liberated in intraventricular hemorrhage (IVH) in preterm infants, which is a frequent complication of prematurity [25, 26]. The incidence of IVH is 15–20% in very low birth weight (<1500 g) preterm infants and even higher (~45%) in extremely low birth weight infants (500–750 g) [27–29]. IVH is associated with high neonatal mortality (20–50%) and increases the risk of neurodevelopmental impairment in surviving infants in addition to the risk associated with prematurity alone [30]. In preterm infants, IVH results from bleeding of the germinal matrix because its capillary network is extremely fragile and unable to regulate cerebral blood flow [31, 32]. IVH leads to systemic inflammation characterized by elevated levels of proinflammatory cytokines, such as TNF, IL-8, and Il-1β; chemokines, such as monocyte adhesion molecule-1; and soluble adhesion molecules (VCAM1 and ICAM1) [33]. As a sign of the local inflammatory response, the levels of soluble adhesion molecules were found to be elevated in the cerebrospinal fluid of patients after subarachnoid hemorrhage [34].

The dark color of the cerebrospinal fluid of premature infants along with our previous finding might indicate the formation of hemoglobin oxidation products, which are a mixture of globin- and porphyrin-centered radicals and covalently cross-linked hemoglobin multimers [15, 35]. These reactions result in the irreversible oxidation of amino acids, which leads to the collapse of α and β subunits [36–38]. Previously, the presence of α- and β-globin chains was described in tissues other than red blood cells, including human brain and peripheral tissues, which suggests that globins or derived peptide fragments have additional physiological functions [39–41]. These findings and a profound examination of the process of hemoglobin oxidation raised the question of whether hemoglobin-derived peptide fragments have effects similar to those of ferryl hemoglobin.

The goal of this study was to identify peptides generated during hemoglobin oxidation in complicated atherosclerotic lesions and intraventricular brain hemorrhage and to investigate whether such peptides provoke endothelial dysfunction. Furthermore, we confirmed the accumulation of ferryl hemoglobin in brain hemorrhage, as previously shown in complicated atherosclerotic lesions.

## Materials and methods

All chemicals were of analytical grade and purchased from Sigma-Aldrich (St. Louis, USA) unless stated otherwise.

### Study approval

Carotid arteries from patients who underwent carotid endarterectomy were obtained from the Department of Surgery of the University of Debrecen. Their collection was approved by the Scientific and Research Ethics Committee of the Scientific Council of Health of the Hungarian Government under registration number DE OEC RKEB/IKEB 3712-2012. The specimens were examined and classified by a pathologist according to the AHA’s criteria. Type I (healthy), type IV (atheromatous), and type VI (complicated) lesions were selected for further investigation. Cerebrospinal fluid samples from premature infants were obtained from the Department of Pediatrics of the University of Debrecen. Their collection was approved by the Scientific and Research Ethics Committee of the Scientific Council of Health of the Hungarian Government under registration number DE RKEB/IKEB 4818-2017.

### Histological staining

Formalin-fixed, paraffin-embedded samples were prepared with a routine procedure. The carotid samples (healthy (*n* = 15), atheromatous lesion (*n* = 12), and complicated plaque with hemorrhage (*n* = 22)) were fixed with a 4% solution of formaldehyde buffered with purified bovine serum at pH 7.4 for a duration of 2–3 days depending on the size of the sample. Buffer (1.0 mol/L EDTA/Tris, pH 7.4, ab64216, Abcam, Cambridge, UK) was used to decalcify the plaques. The samples were dehydrated with an ascending alcohol series, dewaxed with xylol to improve penetration and embedded in paraffin. Sample sections (4-µm thick) were deparaffinized using xylol and ethanol. After the inhibition of endogenous peroxidase activity (3% H_2_O_2_ for 5 min), slides underwent antigen retrieval in a buffered solution (pH 9.0, RE7119, Leica, Wetzlar, Germany). Then, the slides were washed with EnVision FLEX wash buffer (a Tris-buffered saline solution containing Tween 20), pH 7.6 (±0.1) (K8007, Dako, Agilent Technologies, USA). Next, the consecutive sections were incubated with goat antihuman hemoglobin antibody (ab19362-1, Abcam plc, Cambridge, UK) diluted 1:200 for 1 h. Antibody binding was detected by the incubation of specimens with EnVision FLEX/HRP (DM822, Dako, Agilent Technologies, USA) for 30 min in a humidified chamber. The specimens were then reacted with DAB solution for 1–10 min (EnVision FLEX DAB + Chromogen, DM827, Dako, Agilent Technologies). Hematoxylin and eosin staining of the tissue sections was performed with Gill’s hematoxylin solution (105175 Merck Millipore, USA), followed by counterstaining with eosin. For digital documentation, the stained specimens were scanned with a Mirax-Midi scanner (3D Histech, Budapest, Hungary). Macroscopic pictures of the arteries were taken with a Nikon D3200 camera (Nikon Corp.; Minato, Tokyo, Japan).

### Cell culture

Human umbilical vein endothelial cells (HUVECs) were removed by exposure to dispase and cultured in medium 199 containing 15% FBS, antibiotics, l-glutamine, sodium pyruvate, and endothelial cell growth factor as described previously [2]. HUVECs were used at passages 2 and 3 within 2 days after reaching confluence.

### Western blot analysis

For western blot analysis, healthy arteries (*n* = 9), atheromatous plaques (*n* = 9), and hemorrhaged complicated lesions (*n* = 9) were homogenized in liquid nitrogen. Then, the samples were solubilized in protein lysis buffer (10 mmol/L Tris-HCl, 5 mmol/L EDTA, 150 mmol/L NaCl, pH 7.2) containing 1% Triton X-100, 0.5% Nonidet P-40, and protease inhibitors (Complete Mini, F. Hoffmann-La Roche, Ltd, Switzerland). Samples containing 20 µg of protein were loaded onto SDS-PAGE (12.5%) gels. For healthy (*n* = 3) and hemorrhaged brain tissues (*n* = 3), samples containing 20 µg of protein were loaded onto SDS-polyacrylamide (12.5%) gels. For cerebrospinal fluid samples (healthy (*n* = 9), early-phase IVH (*n* = 9), late-phase IVH (*n* = 9)), samples containing 4 µg of protein were loaded onto SDS-polyacrylamide (12.5%) gels. In other cases, cells were cultured in six-well plates, and upon reaching confluence, the cells were treated with different stimuli. After treatment (7 h), the cells were solubilized in protein lysis buffer. Proteins were applied to 12.5% SDS-PAGE gels. After electrophoresis, the proteins were transferred to a nitrocellulose membrane (Amersham Biosciences Corp., USA). Proteins were identified using the following antibodies: mouse antihuman GAPDH (NB-300-221, Novus Biologicals, diluted 1:1000), HRP-conjugated goat antihuman hemoglobin antibody (ab19362-1, Abcam, diluted 1:5000), rabbit antihuman NF-kβ antibody (8242, Cell Signaling Technology, diluted 1:1000), mouse human caspase-1 (CASP1) antibody (MAB6215, R&D Systems, diluted 1:1000), mouse NLRP3/NALP3 human antibody (ALX-804-819-C100, Enzo, diluted 1:1000), rabbit antihuman Lamin B1 antibody (12987-1-AP, Proteintech, diluted 1:500), and mouse antihuman Interleukin 1 Beta (IL-1-β) antibody (abx131957, Abbexa, Ltd, diluted 1:1000). Antigen–antibody complexes were detected with a chemiluminescence system according to the manufacturer’s instructions (GE Healthcare Life Sciences, Piscataway, NJ, USA). Signals were quantified using ImageJ software.

### Hemoglobin separation and purification

Oxyhemoglobin (Fe^2+^), methemoglobin (Fe^3+^), and ferryl hemoglobin were prepared as described before [2, 14]. Hemoglobin was prepared from fresh blood drawn from healthy volunteers using ion-exchange chromatography on a DEAE Sepharose CL-6B column. Methemoglobin prepared by incubation of purified hemoglobin with a 1.5-fold molar excess of K_3_[Fe (CN)_6_] for 30 min at room temperature. Ferryl hemoglobin was made by incubation of oxyhemoglobin with a 10:1 ratio of H_2_O_2_ to heme for 5 h at 37°C. After oxidation, ferryl hemoglobin was dialyzed against saline (three times for 1 h at 4°C) and concentrated with Amicon Ultra centrifugal filter tubes (10,000 MWCO, Merck KGaA, Darmstadt, Germany). Aliquots were snap-frozen in liquid nitrogen and kept at −70 °C. The purity of hemoglobin preparations was assessed by SDS-PAGE, followed by silver staining. Hemoglobin concentrations were calculated as described by Winterbourn [3]. In the present study, the hemoglobin concentration is always expressed as the heme concentration.

### Hemoglobin oxidation

Hemoglobin (10 µmol/L) was oxidized with H_2_O_2_ (40 µmol/L) at room temperature for 30 min. To assess the effect of haptoglobin on the fragmentation of hemoglobin, hemoglobin was incubated with haptoglobin (Hp 1-1, 330-12, Lee BioSolutions, 10 µmol/L) and then exposed to H_2_O_2_ (40 µmol/L) at room temperature. The oxidized products were concentrated with an Amicon Ultra-0.5 centrifugal filter unit with an Ultracel-10 membrane (UFC501096, Merck KGaA, Darmstadt, Germany). The concentrated fraction (containing 30 µg of protein) was subjected to SDS-PAGE (NW04120BOX, NuPAGE Bis-Tris Precast Gel, Thermo Scientific).

### Coomassie brilliant blue staining

For tissue analysis, samples (healthy arteries (*n* = 6), atheromatous plaques (*n* = 5), and hemorrhaged complicated lesions (*n* = 10)) were homogenized in liquid nitrogen. For cerebrospinal fluid analysis, healthy (*n* = 7), early-phase IVH (*n* = 6), and late-phase IVH (*n* = 6) samples were collected. After electrophoresis, the gel was fixed in fixing solution (50% methanol and 10% glacial acetic acid) for 1 h and incubated in staining solution (0.1% Coomassie Brilliant Blue R-250, 50% methanol and 10% glacial acetic acid) for 45 min with shaking. The gel was destained with destaining solution (40% methanol and 10% glacial acetic acid) until no background staining was visible to visualize protein bands.

### Cross-linking mass spectrometry

Haptoglobin (Hp 1-1, 330-12, Lee BioSolutions), serum albumin (CSL Behring GmbH, 04047725118975) and hemoglobin-derived peptides (Peptide 1–Peptide 5) were prepared in 10 µmol/L PBS (pH 7.0). The cross-linker DSSO (A33545, Thermo Scientific) was dissolved in DMSO (2025920, Invitrogen) at a final concentration of 50 mmol/L. Cross-linking reactions were carried out at room temperature for 1 h with continuous shaking by using DSSO at a final concentration of 10 µmol/L. Samples were processed with the GeLC method [42] and digested with trypsin. The digested peptides were extracted from the gel and subjected to LC–MS/MS analyses. Peptides were separated using an Easy nLC1200 nanoUPLC system (Thermo Scientific) with a 60 min water–acetonitrile gradient. First, desalting was carried out on an Acclaim PepMap C18 nano trap column (20 × 75 µm, 3 μm particle size, 100 Å pore size, Thermo Scientific), followed by separation on an Acclaim PepMap RSLC analytical column (150 mm × 50 μm, 2 μm particle size, 100 Å pore size, Thermo Scientific). Solvent A was 0.1% formic acid in LC water, and solvent B was 0.1% formic acid in LC acetonitrile. The flow rate was set to 300 Nl/min. Mass spectrometry analyses were performed on an Orbitrap Fusion Tribrid mass spectrometer (Thermo Scientific) in data-dependent mode. During the scan, the 14 most intensive ions in the Orbitrap analyzer (resolution: 60,000, AGC target: 4.0e5) were selected for CID fragmentation (CID energy: 30%), and the fragments were analyzed in the linear ion trap (AGC target: 2.0e3). Protein identification against the sequences of haptoglobin, albumin, and hemoglobin-derived peptides was carried out based on registered MS/MS spectra using the MaxQuant 1.6.2.10 search engine [43]. Cysteine carbamidomethylation, methionine oxidation, and N-terminal acetylation were set as variable modifications. The results of MaxQuant searches were imported to Scaffold 4.8.9 software (Proteome Software, Inc). Using a 1% FDR threshold at the protein level and a 0.1% FDR threshold at the peptide level, we successfully identified haptoglobin and albumin in the samples. Possible cross-links between proteins and the hemoglobin-derived peptides were analyzed using MeroX 2.0 software [44, 45].

### Sample preparation for mass spectrometry

A 16 kDa band and the gel bands below were excised, and after destaining, the proteins were reduced for 1 h at 56 °C with 20 mmol/L dithiothreitol and then alkylated for 45 min in the dark with 55 mmol/L iodoacetamide. Overnight trypsin digestion was carried out using MS-grade stabilized bovine trypsin (SCIEX) at 37 °C, and the digested peptides were extracted from the gels and dried. The peptides were redissolved in 10 μl of 1% formic acid before LC–MS/MS analysis.

### LC–MS/MS analysis

Prior to mass spectrometry analysis, the peptides were separated using an Easy-nLCII (Bruker) nano HPLC with a 90 min water/acetonitrile gradient with an increasing acetonitrile concentration from 0% to 100% over 60 min. First, the peptide mixture was desalted on a Zorbax 300SB-C18 in-line trap column (5 × 0.3 mm, 5 μm particle size, Agilent), followed by separation on a Zorbax 300SB-C18 analytical column (150 mm × 75 μm, 3.5 μm particle size, Agilent). Solvent A was 0.1% formic acid in LC water, and solvent B was 0.1% formic acid in LC acetonitrile. The flow rate was set to 300 Nl/min. Positive LC–MS/MS scans were performed on a 4000 QTRAP (ABSciex) mass spectrometer using a NanoSpray II MicroIon source controlled by Analyst 1.4.2 software (ABSciex). The spray voltage was 2800 V, the curtain gas pressure was 20 psi, the ion source gas pressure was 50 psi, and the source temperature was set to 70 °C. For MS/MS analysis, the information-dependent acquisition method was utilized. After the first mass scan (mass range 400–1700 amu), an enhanced resolution scan was carried out to establish the charge states of the precursor ions. The MS/MS spectra of the two most intensive ions were recorded (mass range 100–1900 amu) in enhanced product ion mode at a scan rate of 4000 amu/s and rolling collision energy was applied with a maximum of 80 eV. The acquired LC–MS/MS spectra were used for protein identification with the help of the Protein Pilot 4.0 (ABSciex) search engine searching the SwissProt database (release: 2015.12, 550,116 sequence entries) and the biological modification table included in the Protein Pilot 4.0 software. Peptide identification was validated manually; 90% confidence and the presence of four b or y ions in series were the minimum criteria.

### Peptides

Synthetic, purified hemoglobin peptides (VNVDEVGGEALGRLLVVYPWTQR, LLVVYPWTQR, MFLSFPTTK, VGAHAGEYGAELERMFLSFPTTK, and FLASVSTVLTSKYR) were purchased from JPT (Germany). The peptides were aliquoted and stored at −20 °C until use.

### Endothelial cell monolayer integrity assay

Cells were cultured on eight-well electrode arrays (8W10E, Applied BioPhysics, Inc., USA) and upon reaching confluence, cells were treated with hemoglobin (10 μmol/L), ferryl hemoglobin (10 μmol/L), and peptides 1–5 (100 pmol/L, 10 μmol/L). ECIS Z-Theta instrument (Applied BioPhysics Inc., USA) was applied to monitor the complex impedance spectrum for 7 h. Intercellular gap formation was calculated based on the difference between monolayer resistance at 32 kHz at the 0 h time point and at 7 h.

### Cell viability assay

Cells were cultured in 96-well plates, and upon reaching confluence, the cells were treated with hemoglobin, ferryl hemoglobin and the five peptides (10 µmol/L). After 7 h of incubation, the monolayers were washed twice with HBSS, and the test solutions were replaced with 100 μl of a 3-[4,5-dimethylthiazol-2-yl]-2,5-diphenyl-tetrazolium bromide (MTT) (0.5 mg/mL) solution in HBSS, followed by an additional 3 h of incubation. After the MTT solution was removed, 100 μl of dimethyl sulfoxide (DMSO) was added to the wells, and the optical density at 570 nm was measured.

### Cell nuclear protein preparation

Cells were cultured in six-well plates, and upon reaching confluence, the cells were treated with different stimuli. After treatment (1.5 h), 500 µl of buffer A (20 mmol/L Tris, pH 7.5–8.0, 100 mmol/L NaCl, 300 mmol/L sucrose, 3 mmol/L MgCl_2_) was added per well, and the cells were scraped thoroughly and then incubated on ice for 10 min. The samples were centrifuged at 4 °C at 3000 rpm for 10 min. Then, the supernatant was removed. The pellet was resuspended on ice in 374 µl of buffer B (20 mmol/L Tris, pH 8.0, 100 mmol/L NaCl, 2 mmol/L EDTA, pH 8.0), and 26 µl of 4.6 mol/L NaCl was added. Then, the samples were homogenized with 20 full strokes in a Dounce or glass homogenizer on ice. After incubation on ice for 30 min, the samples were centrifuged at 24,000 × *g* for 20 min at 4 °C. The supernatant was aliquoted, and 10 µl was removed to measure the total protein concentration.

### Immunoprecipitation of carotid artery samples

Pierce protein A/G magnetic agarose beads (40 µl) were added to a 1.5 ml microcentrifuge tube. A total of 460 μl of binding/wash buffer (10 mmol/L phosphate buffer (pH 7.4), 150 mmol/L NaCl) was added to the beads. The tube was placed into a magnetic stand to collect the beads against the side of the tube. The supernatant was removed and discarded. Binding/wash buffer (0.5 ml) was added to the tube and gently mixed for 1 min. The beads were collected with the magnetic stand, and the supernatant was removed and discarded. Primary antibody at a tenfold higher concentration than that used for western blotting was added to a total volume of binding/wash buffer of 500 μl. The antibody-bead mixture was incubated for 4 h at 4 °C by gentle mixing on a suitable shaker. The tube was placed into a magnetic stand to collect the beads against the side of the tube, and the supernatant was removed and discarded. Binding/wash buffer (0.5 ml) was added to the tube and gently mixed for 1 min. The washing step was repeated twice. Fifty microliters of tissue lysate was added with 450 μl of binding/wash buffer to a 1.5 ml microcentrifuge tube. The lysate-bead/antibody conjugate mixture was incubated at 4 °C under rotary agitation overnight. The beads were washed three times with binding/wash buffer containing a 1:20 volume of protease inhibitor. The tube was placed into a magnetic stand to collect the beads against the side of the tube, and the supernatant was removed and discarded. One-hundred microliters of elution buffer (0.1 M glycine, pH 2.0–3.0) was added to the tube and incubated for 10 min at room temperature with occasional mixing. The beads were collected with a magnetic stand, and the supernatant was then removed and saved. To neutralize the low pH, 100 μl of neutralization buffer (1 mol/L Tris-HCl, pH 7.5) was added for each 100 μl of eluate. Western blotting was used to assess protein precipitation.

### Monocyte adhesion assay

Human endothelial cells were cultured on coverslips in 24-well plates. Upon reaching confluency, the cells were treated with Peptides 1–5 and ferryl hemoglobin for 6 h. Human blood-derived monocytes were collected from the blood of healthy donors. Phase centrifugation with Histopaque-1077 was used to separate monocytes. Mononuclear cells were suspended in serum-free Dulbecco’s Modified Eagle Medium. Then, mononuclear cells were incubated with calcein-AM for 30 min at 37 °C. Labeled monocytes (2 × 10^5^ cells/well) were added to human endothelial cells in complete culture medium and incubated for 30 min at 37 °C. After that, the cells were fixed with 3.7% formaldehyde.

### Immunofluorescence staining

Cells were cultured on coverslips in 24-well plates. Upon reaching confluence, cells were challenged with hemoglobin, ferryl hemoglobin, and Peptides 1–5 for 7 h. Cells were fixed with 3.7% formaldehyde for 15 min. F-Actin was stained with iFluor 647 (ab176759, Abcam), Hoechst (33258) was used to stain nuclei. To show NF-kβ nuclear translocation, cells were challenged with Peptides 1–5, hemoglobin, and ferryl hemoglobin for 1.5 h. After treatment, the cells were fixed with 3.7% formaldehyde for 15 min. After fixation, the cells were blocked with 5% goat serum for 1 h at room temperature. Rabbit monoclonal antihuman NF-kβ (701079, Thermo Scientific, diluted 1:25) was used as a primary antibody to show NF-kβ nuclear translocalization in endothelial cells. AF488-labeled goat anti-rabbit secondary antibody (A11070, Thermo Scientific, diluted 1:500) was incubated with the cells for 1 h in the dark at room temperature. F-Actin was stained with iFluor 647 (ab176759, Abcam). Hoechst (33258) was used to stain nuclei.

### STED nanoscopy

Multicolor STED images were acquired with Leica TCS SP8 STED (Stimulated Emission Depletion) system (Leica Microsystem Mannheim, Germany). Gated STED images were deconvoluted using Huygens Professional software (Scientific Volume Imaging B.V., Hilversum, Netherlands).

### Quantitative real-time PCR (qRT-PCR)

Human umbilical endothelial cells were treated as indicated before. Total RNA was isolated using RNAzol STAT-60 according to the manufacturer’s instructions (Tl-4120, Tel-test, Inc., USA). RNA concentrations were measured with Implen N50 nanophotometer (Implen GmbH, München, Germany). Then, cDNA synthesis was done with the cDNA kit (43-688-13, Applied Biosystems, USA). Real-time PCR was used to quantify the mRNA levels of IL-1β, TNF, ICAM1, and GAPDH. All primers were purchased from Thermo Fisher Scientific. TaqMan Universal PCR Master Mix was purchased from Applied Biosystems (4269510, Applied Biosystems). Finally, we performed TaqMan quantitative PCR with a Bio-Rad CFX96 detection system (Bio-Rad Laboratories, USA). The results are expressed as mRNA expression and were normalized to GAPDH mRNA expression.

### Statistical analysis

Data were analyzed by GraphPad Prism 5.02 software (GraphPad Software, CA, USA). Data are shown as the mean ± SD. Statistical analysis was performed by one-way ANOVA followed by Dunnett’s or unpaired *t*-test. Differences for which *P* < 0.05 were considered significant.

## Results

### Oxidation of hemoglobin generates peptide fragments in both atherosclerotic lesions and intraventricular hemorrhage of the human brain

**Fig. 1:**
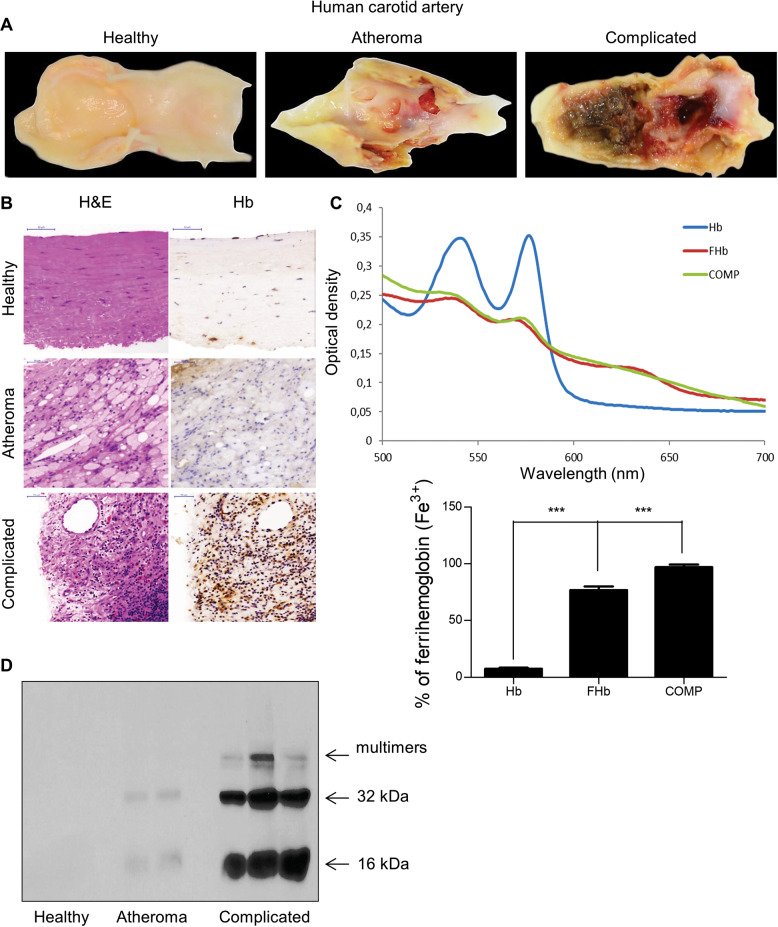
Hemoglobin is oxidized in ruptured atherosclerotic lesions, generating ferryl hemoglobin. Macroscopic images (**a**), histopathological analysis with hematoxylin and eosin (H&E) staining and immunohistological analysis with anti-hemoglobin (Hb) antibody of the human carotid artery: healthy arterial intima, atheromatous lesion, and complicated plaque with hemorrhage (**b**). Spectral analysis of hemoglobin (Hb), ferryl hemoglobin (FHb), and complicated plaque (Comp); the percentage of oxidized hemoglobin was calculated (**c**). Representative western blot of the human carotid artery using anti-Hb antibody (*n* = 3) (**d**). The results are shown as the mean values ± SEMs of the experiments. *P < 0.05, **P < 0.01, ***P < 0.001.

We previously described the oxidation of hemoglobin and the formation of ferryl hemoglobin in human atherosclerotic lesions [9]. Drawing upon our findings, we followed the fate of the globin chain of hemoglobin within these lesions. Figure 1 shows macroscopic and microscopic images of carotid arteries derived from human subjects treated with endarterectomy. The accumulation of lipids in atheromatous plaque and intraplaque hemorrhage in complicated lesions are shown in Fig. 1a, b, respectively. Hematoxylin and eosin (H&E) staining confirmed extracellular and intracellular intimal fat accumulation in atheroma and the presence of red blood cells in complicated lesions. The accumulation of free hemoglobin within the plaque was revealed by immunohistochemistry (Fig. 1b). Spectral analysis of hemoglobin derived from complicated lesions indicated its oxidation and the generation of ferrihemoglobin (Fig. 1c). In the case of ferrohemoglobin, the curve shows two absorption peaks at 540 and 576 nm, while the curve for the complicated plaque is similar to a typical ferrihemoglobin spectrum, with peaks at 535, 575, and 630 nm. As shown in Fig. 1d, the formation of dimers, tetramers, and multimers of hemoglobin in complicated lesions is a hallmark of ferryl hemoglobin.

**Fig. 2:**
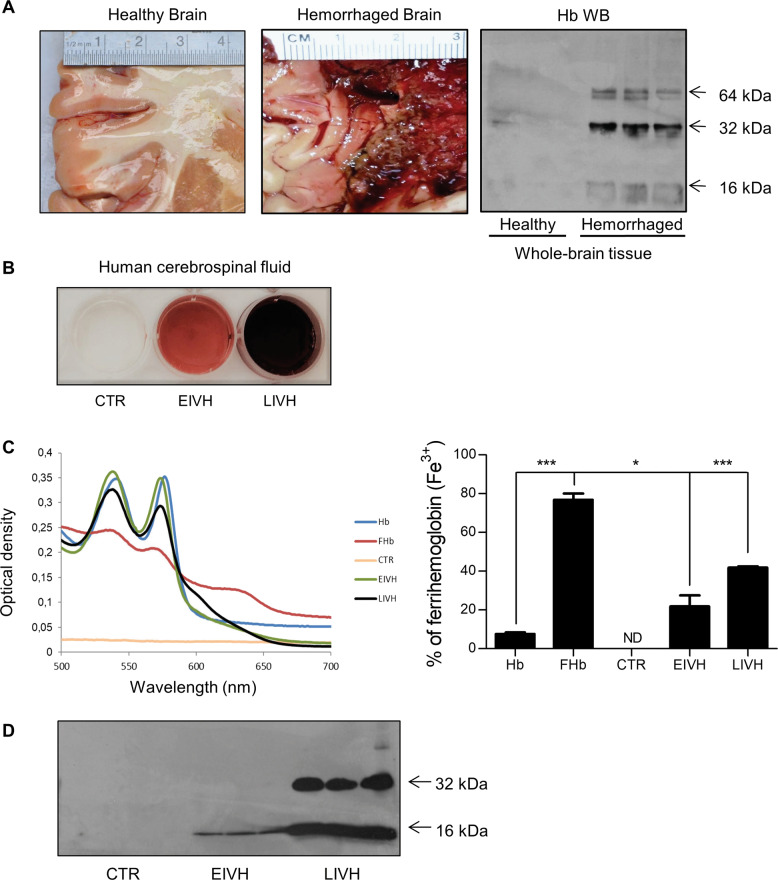
Hemoglobin is oxidized in intraventricular hemorrhage of the brain, generating ferryl hemoglobin. Macroscopic images of healthy and hemorrhaged brain and western blot analysis of healthy and hemorrhaged tissues using anti-hemoglobin antibody (**a**). Human cerebrospinal fluid: healthy (CTR), early-phase intraventricular hemorrhage (EIVH), and late-phase intraventricular hemorrhage (LIVH) (**b**). Spectral analysis of Hb, FHb, CTR cerebrospinal fluid, EIVH cerebrospinal fluid, and LIVH cerebrospinal fluid. The percentage of oxidized hemoglobin was calculated. ND indicates not detectable (**c**). Western blot analysis of cerebrospinal fluid samples with anti-hemoglobin antibody (**d**). The immunoblot is representative, *n* = 3. The results are shown as the mean values ± SEM of the experiments. *P < 0.05, **P < 0.01, ***P < 0.001.

Since hemoglobin has been implicated in the pathophysiology of brain hemorrhage [46], we also examined the formation of ferryl hemoglobin in the hemorrhaged brain and hemorrhaged cerebrospinal fluid. Macroscopic images of healthy and hemorrhaged brains from cadavers are presented in Fig. 2a. Western blot analysis revealed the presence of dimers, tetramers, and multimers of hemoglobin in hemorrhaged brains. Representative images of healthy hemorrhaged cerebrospinal fluid (early- and late-phase) taken from premature infants are shown in Fig. 2b. Spectrum analysis of these samples indicated the oxidative modification of hemoglobin. The absorption curve of hemoglobin in early-phase IVH exhibited peaks at 540 and 576 nm, indicating the presence of ferrohemoglobin (Fig. 2c). In contrast, a decrease in the signal at 535 and 575 nm accompanied by the appearance of a new peak at 600 nm, which indicates the presence of ferrihemoglobin, was noted in the absorption curve of hemoglobin in late-phase IVH (Fig. 2c). Importantly, the oxidation of hemoglobin was accompanied by the formation of cross-linked hemoglobin. In hemorrhaged cerebrospinal fluid in late-phase IVH, hemoglobin dimers were found, indicating the formation of ferryl hemoglobin, unlike hemorrhaged cerebrospinal fluid in early-phase IVH, in which monomers were only present (Fig. 2d).

**Fig. 3:**
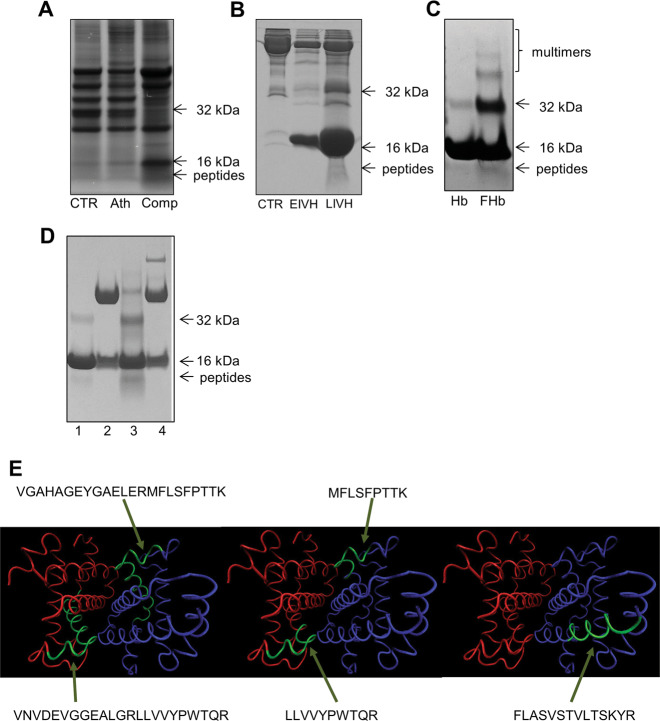
Oxidation of hemoglobin in ruptured atherosclerotic lesions and intraventricular hemorrhage of the human brain generates peptide fragments. Healthy (CTR), atheromatous lesion (Ath), and complicated lesion (Comp) human carotid artery samples were subjected to SDS-PAGE and stained with Coomassie Brilliant Blue R-250 (*n* = 3) (**a**). Cerebrospinal fluid: healthy (CTR), early-phase intraventricular hemorrhage (EIVH), and late-phase intraventricular hemorrhage (LIVH) samples were subjected to SDS-PAGE and stained with Coomassie Brilliant Blue R-250 (**b**). After the oxidation of hemoglobin with hydrogen peroxide (FHb), the sample was subjected to SDS-PAGE and stained with Coomassie Brilliant Blue R-250 (**c**). Hemoglobin (1), haptoglobin (2), FHb (3), and hemoglobin incubated with haptoglobin and hydrogen peroxide (4) were subjected to SDS-PAGE and stained with Coomassie Brilliant Blue R-250 (**d**). Structure of hemoglobin according to the 3onz.pdb crystal structure deposited in the Protein Data Bank (**e**); the sequences of the peptides whose biological effects were examined are indicated, and the arrows show their position in the 3D structure of hemoglobin. Blue indicates chain A, red indicates chain B, and green indicates the peptides.

As we examined hemoglobin on SDS-PAGE gels stained with Coomassie Brilliant Blue, bands below 16 kDa, consisting of hemoglobin samples were noted in samples of both complicated atherosclerotic plaque and the cerebrospinal fluid in late-phase IVH (Fig. 3a, b). To identify the bands below 16 kDa, they were subjected to peptide identification by LC–MS/MS-based mass spectrometry. The identified peptides were matched with hemoglobin sequences.

Based on the results of peptide examination, the most prominent hemoglobin-derived peptides identified in different samples were LLVVYPWTQR, which was observed 15 times, and MFLSFPTTK, which was observed 13 times, (Supplementary Table). Because we used trypsin as the cleavage enzyme during sample preparation for mass spectrometry, these identified peptides are tryptic peptides. However, the available information on the identified tryptic peptides did not indicate the exact length of the native peptides. To gain insight into the effect of longer peptides in addition to the most prominent ones, we arbitrarily chose longer forms of the LLVVYPWTQR and MFLSFPTTK peptides: VNVDEVGGEALGRLLVVYPWTQR and VGAHAGEYGAELERMFLSFPTTK, respectively. To determine whether a less abundant peptide has biological effects as well, from the less abundant peptides, we arbitrary chose FLASVSTVLTSKYR, which was observed only four times during peptide identification (Supplementary Table).

To gain information on the origin and nature of these peptides and determine whether they are the result of the oxidative processes of hemoglobin, we exposed hemoglobin to H_2_O_2_. After electrophoresis and Coomassie staining, the most intensive band at 16 kDa indicated the presence of hemoglobin monomers, and bands above 16 kDa corresponding to hemoglobin dimers, trimers, tetramers (Fig. 3c), which are hallmark of ferryl hemoglobin formation, appeared. A less intensive band below 16 kDa was also detected. By employing LC–MS/MS-based mass spectrometry, we identified peptide fragments (Supplementary Table). To examine whether haptoglobin alters the fragmentation of hemoglobin provoked by peroxide, we incubated hemoglobin with haptoglobin and then exposed the sample to H_2_O_2_. As shown in Fig. 3d, no bands below 16 kDa in the presence of haptoglobin were detected after electrophoresis and Coomassie staining, indicating an inhibitory effect.

To explore the potential vasculopathic impact of hemoglobin peptides, we employed five pure peptides in our further experiments: VNVDEVGGEALGRLLVVYPWTQR (labeled Peptide 1), LLVVYPWTQR (labeled Peptide 2), MFLSFPTTK (labeled Peptide 3), VGAHAGEYGAELERMFLSFPTTK (labeled Peptide 4), and FLASVSTVLTSKYR (labeled Peptide 5) (Fig. 3e).

### Haptoglobin and albumin do not bind peptide fragments resulting from hemoglobin oxidation

Since the fragmentation of hemoglobin occurs in both complicated atherosclerotic lesions and IVH of the brain, we assessed whether peptides resulting from hemoglobin oxidation bind haptoglobin or albumin using cross-linking mass spectrometry. No cross-links between the albumin- and hemoglobin-derived peptides or haptoglobin and peptides P1–P5 were identified.

### Peptides identified from hemorrhaged atherosclerotic plaques and cerebrospinal fluid induce intercellular gap formation and ***decrease*** junctional ***resistance*** in the endothelium

**Fig. 4:**
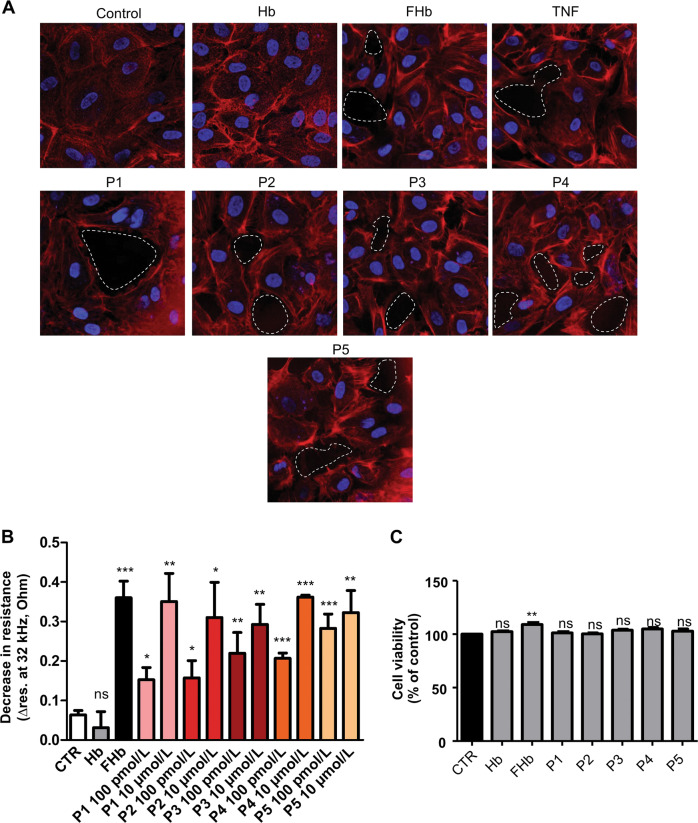
Intercellular gap formation and decreased endothelial impedance in response to peptides derived from hemoglobin oxidation. Confluent human umbilical vein endothelial cells (HUVECs) were exposed to Hb (10 µmol/L), FHb (10 µmol/L), TNF and peptides (P1–P5) (10 µmol/L) as described in the “Methods” section. Cells were stained for DNA (Hoechst 33258, blue) and F-actin (cytoskeleton, red, iFluor 647). Dashed white circles indicate intercellular gaps. Representative staining, *n* = 5 (**a**). Bioimpedance was measured with ECIS at 32 kHz. HUVECs were exposed to Hb (10 µmol/L), FHb (10 µmol/L), and peptides (100 pmol/L and 10 µmol/L) (*n* = 5) (**b**). The results are shown as the mean values ± SEM of the experiments. *P < 0.05, **P < 0.01, ***P < 0.001, ns means not significant. An MTT assay was performed to determine toxicity. HUVECs were treated as mentioned above. Data are the means ± SEMs of four separate experiments repeated as 12 technical replicates. ***P < 0.05 (**c**). Control (CTR) means untreated.

Ferryl hemoglobin was previously demonstrated to activate endothelial cells in vitro, which leads to the formation of intercellular gaps [14]. Therefore, we tested whether peptides derived from hemoglobin oxidation have a similar effect. Endothelial cells were exposed to the five most prominent peptides identified from hemorrhaged atherosclerotic plaques and cerebrospinal fluid to hemoglobin, ferryl hemoglobin, and TNF [47]. As demonstrated by fluorescence microscopy, all five peptides rearranged the actin cytoskeleton and led to the formation of intercellular gaps that disrupted the integrity of the endothelial cell monolayer (Fig. 4a). Simultaneously, we also examined how peptides and ferryl hemoglobin affect junctional resistance between endothelial cells. Impedance measurements were performed using electric cell-substrate impedance sensing (ECIS) (Fig. 4b). As shown in Fig. 4b, a dose-dependent decrease in the impedance of the endothelium was observed after exposure to the peptides, although the extent of this change in impedance varied significantly between the five peptides. Peptide 5 had the most pronounced effect, while Peptides 1 and 2 provoked the least pronounced but still significant disruption.

We assessed cell viability by performing an MTT assay after exposure of the endothelium to hemoglobin, ferryl hemoglobin, and peptides. As demonstrated in Fig. 4c, viability of cells exposed to peptides derived either alpha or beta chain did not decline.

### Peptides resulting from hemoglobin oxidation enhance monocyte adhesion to endothelial cells

**Fig. 5:**
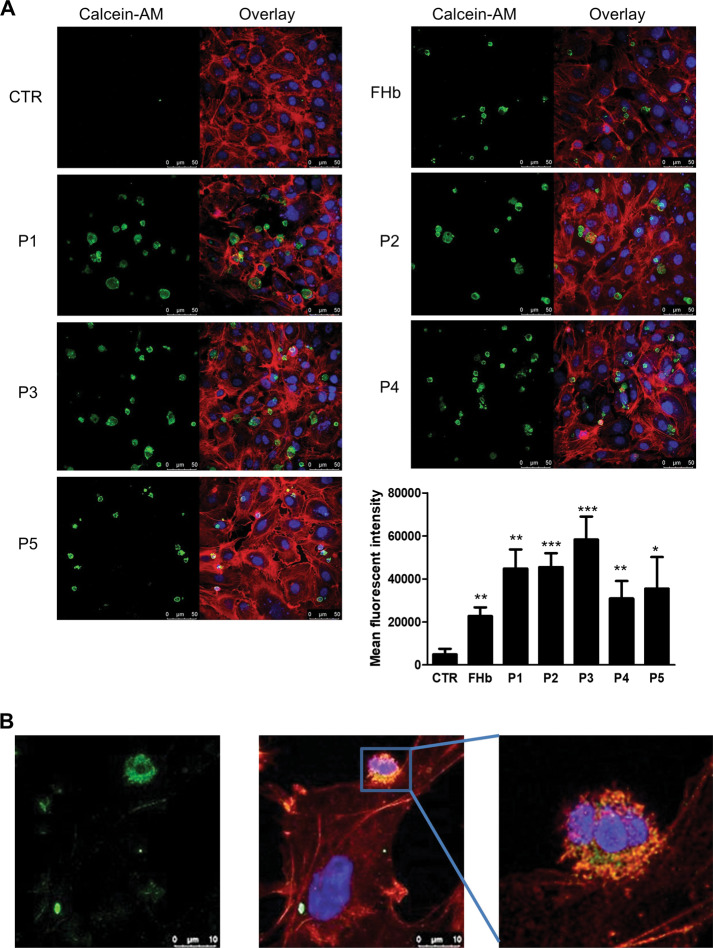
Increased monocyte adhesion to the endothelium in response to hemoglobin-derived peptides and ferryl hemoglobin. Adhesion of monocytes to HUVECs after exposure to FHb (10 µmol/L) and peptides (P1–P5) (10 µmol/L). HUVECs were stained for DNA (Hoechst 33258, blue) and F-actin (cytoskeleton, red, iFluor 647). Monocytes were stained with calcein-AM. The mean fluorescence intensity is shown (**a**). Magnified images demonstrating monocyte adhesion to endothelial cells after Peptide 3 treatment (B). Representative images, *n* = 5. The results are shown as the mean values ± SEMs of the experiments. *P < 0.05, **P < 0.01, ***P < 0.001. CTR means untreated.

Since ferryl hemoglobin enhances monocyte adhesion to the endothelium [15], we asked whether hemoglobin-derived peptides also increase monocyte adhesion to vascular endothelial cells. As shown in Fig. 5a, no monocyte binding to confluent untreated endothelial cells was observed. In contrast, adhesion was observed when endothelial cells were exposed to the five peptides and ferryl hemoglobin. As demonstrated in Fig. 5, all five peptides had a significant effect, although the extent of this effect varied. Particularly, Peptides 2 and 3 increased monocyte adhesion to the endothelium to the greatest extent. The response of tight cell–cell interactions between a monocyte and an endothelial cell in response to Peptide 3 is revealed in Fig. 5b. These findings suggest that peptides derived from hemoglobin oxidation provoke inflammatory responses via activating the endothelium.

### Peptides derived from hemoglobin oxidation activate the transcription factor NF-κβ

**Fig. 6:**
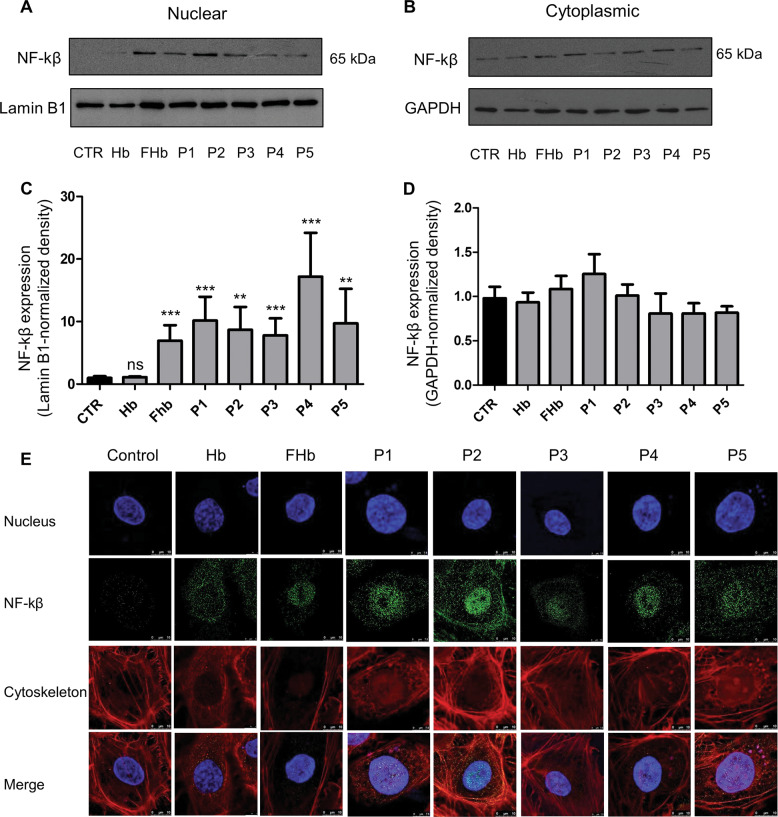
Hemoglobin oxidation activates the transcription factor NF-κβ via hemoglobin-derived peptides and ferryl hemoglobin. NF-kβ levels in the nucleus (**a**) and cytoplasm (**b**) were determined by western blot analyses. The band intensities were normalized to those of Lamin B1 in the case of nuclear extracts (**c**) and GAPDH in the case of cytoplasmic extracts (**d**). Immunoblots are representative of three independent experiments. HUVECs were stained for DNA (Hoechst 33258, blue), NF-kβ (green, Alexa Fluor 488), and F-actin (cytoskeleton, red, iFluor 647). Images were obtained by immunofluorescence-confocal STED nanoscopy (**e**) Representative images, *n* = 5. The results are shown as the mean values ± SEMs of the experiments. *P < 0.05, **P < 0.01, ***P < 0.001, ns means not significant. Control (CTR) means untreated.

Most of the proinflammatory genes expressed during endothelial cell activation are regulated by the NF-κβ signaling pathway [14, 48]. Therefore, we challenged endothelial cells with Peptides 1–5, hemoglobin, and ferryl hemoglobin and assessed the nuclear translocation of NF-κβ. Nuclear accumulation of NF-κβ occurred after cells were exposed to Peptides 1–5, as shown in Fig. 6a–d and determined by western blot analysis. To confirm the presence of NF-κβ in nuclei, we applied STED nanoscopy. In the nuclei of cells treated with Peptides 1–5 as well as ferryl hemoglobin, NF-κβ was observed (Fig. 6e). These observations suggest that the oxidation of hemoglobin is a proinflammatory stimulus as it generates ferryl hemoglobin and peptide fragments via activation of the transcription factor NF-κβ.

### Hemoglobin-derived peptides induce proinflammatory genes

**Fig. 7:**
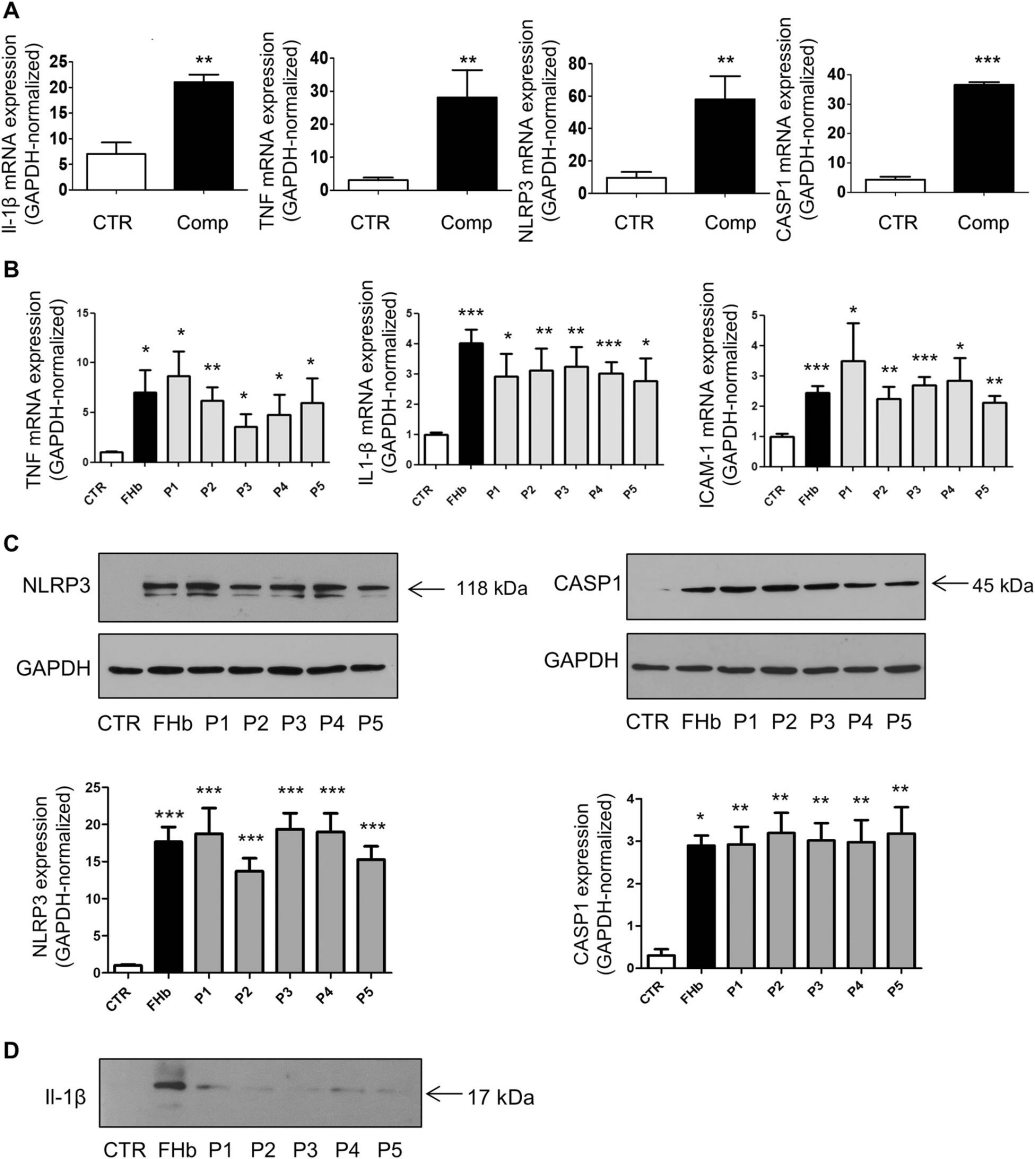
Hemoglobin-derived peptides and ferryl hemoglobin induce proinflammatory gene expression in the endothelium. Relative expression of IL-1β, TNF, NLRP3, and CASP1 from human healthy/complicated carotid arteries was analyzed by real-time qPCR (*n* = 5) (**a**). HUVECs were treated with FHb and the peptides (10 µmol/L). Relative expression of TNF, IL-1-β, and ICAM1 was analyzed by real-time qPCR (*n* = 3) (**b**). Protein expression of NLRP3, CASP1, and GAPDH was determined by western blotting. The band intensities were normalized to that of GAPDH (**c**). Protein expression of Il-1β in the supernatant was determined by western blotting (**d**). Immunoblots are representative, *n* = 3. The results are shown as the mean values ± SEMs of the experiments. *P < 0.05, **P < 0.01, ***P < 0.001. CTR means untreated.

In innate immunity, NOD-like receptor protein 3 (NLRP3) plays an important role through the formation of inflammasomes that activate CASP1, which processes NF-κβ-dependent proinflammatory cytokines, e.g., pro-IL-1β, to their mature forms. To analyze the expression levels of proinflammatory genes in complicated lesions of the human carotid artery, we implemented RT-qPCR of complicated plaque regions. As shown in Fig. 7a, IL-1β, TNF, NLRP3, and CASP1 gene expression was higher in human complicated lesions of the carotid artery than in healthy carotid arteries.

To explore whether exposure to Peptides 1–5 has a proinflammatory effect in endothelial cells, we performed RT-qPCR. We found that after endothelial cells were challenged with Peptides 1–5 and ferryl hemoglobin, the mRNA levels of IL-1β, TNF, and ICAM1 increased (Fig. 7b). Accordingly, Peptides 1–5 and ferryl hemoglobin increased the protein levels of NLRP3 and CASP1, as shown by western blot analysis (Fig. 7c). The protein expression of Il-1β from the cell culture supernatant also increased after the cells were exposed to Peptides 1–5 and ferryl hemoglobin (Fig. 7d).

## Discussion

Hemoglobin is oxidized in both hemorrhaged atherosclerotic lesions and IVH [9, 25]. In this study, we examined the pathophysiologic role of hemoglobin oxidation and the fate of hemoglobin in these pathologies. While hemoglobin is compartmentalized in red blood cells, its oxidation is prevented by a highly effective antioxidant defense system [49]. Outside red blood cells, hemoglobin is prone to oxidation, leading to the formation of different hemoglobin oxidation products and subsequent release of heme [2]. Hemoglobin, the heme iron in which is in the ferrous (Fe^2+^) oxidation state, can undergo one-electron auto-oxidation, leading to the formation of methemoglobin (ferric, Fe^3+^). Peroxides such as H_2_O_2_, can cause the two-electron oxidation of hemoglobin, producing ferryl (Fe^4+^ = O^2−^) hemoglobin, whereas the reaction of methemoglobin with H_2_O_2_ yields ferryl hemoglobin radical [Hb• + (Fe^4+^ = O^2−^)] in which the unpaired electron is located mostly on the globin chains [35, 37, 50–52]. The formed high-valence (ferryl, Fe^4+^) iron compounds are reactive intermediates that decay quickly via intramolecular electron transfer between the ferryl iron and specific amino acid residues of the globin chains, resulting in the formation of globin radicals [53, 54]. Then, termination of the reaction occurs when these globin radicals react, leading to the formation of covalently cross-linked hemoglobin multimers.

Previously, we detected covalently cross-linked hemoglobin forms and the reduced dityrosine content of hemoglobin in human complicated atherosclerotic lesions with intraplaque hemorrhage [9]. In this study, we show for the first time that, similar to their accumulation in complicated atherosclerotic plaques, ferryl hemoglobin/covalently cross-linked hemoglobin multimers accumulate in the cerebrospinal fluid of preterm infants after IVH.

We also observed the accumulation of peptides derived from the oxidation of hemoglobin in both hemorrhaged atherosclerotic plaques and IVH. Previous studies have shown that the oxidation of hemoglobin results in activation of the endothelium via the generation of ferryl hemoglobin [14]. In this study, we asked whether hemoglobin-derived peptides also exhibit proinflammatory effects in endothelial cells.

We identified hemoglobin-derived fragments from atherosclerotic lesions and IVH using LC–MS/MS-based mass spectrometry and then selected the most prominent peptides for further examinations (VNVDEVGGEALGRLLVVYPWTQR, LLVVYPWTQR, MFLSFPTTK, VGAHAGEYGAELERMFLSFPTTK, and FLASVSTVLTSKYR).

Both fluorescence microscopy and bioimpedance measurements revealed that endothelial cells exposed to hemoglobin-derived peptides showed rearrangement of the actin cytoskeleton, resulting in disruption of the endothelial cell monolayer, intercellular gap formation, and increased permeability of the monolayer. The enhanced adhesion of human monocytes to the endothelium in response to peptides was also noted. Endothelial barrier function is a well-regulated process [55] including vascular permeability and leukocyte extravasation. Connective structure that links vascular endothelial cells to each other and to the extracellular matrix participates in leukocyte adhesion, movement, and matrix remodeling. Although peptides derived from hemoglobin oxidation provoked both intercellular gap formation, decreased transcellular electrical resistance, and increased monocyte adhesion to endothelium some caveats are acknowledged. In our experimental conditions the most potent inducer for intercellular gap formation and lowering junctional resistance was Peptide 5. On the contrary, the increase in monocyte adhesion to endothelium was most pronounced after exposure of cells to Peptide 2 and Peptide 3. The difference in the extent of cellular response may resulted from the distinct regulation of monocyte adhesion to endothelium as compared with the regulation of alignment of endothelial cells by the connective structures.

Importantly, we observed the nuclear accumulation of NF-κβ in endothelial cells exposed to peptides. Most of the proinflammatory genes expressed during endothelial cell activation are regulated by the NF-κβ signaling pathway [14, 48]. In resting cells, NF-κβ is inactive and sequestered to the cytoplasm in a complex with the inhibitory protein Iκ-Bα. Iκ-Bα is responsible for masking the nuclear localization sequence of NF-κβ. Upon stimulation, e.g., by IL-1α, TNF or LPS, Iκ-Bα is phosphorylated, and after its degradation, NF-κβ is released and translocates into the nucleus [56, 57].

Previous studies have shown that cytokines are involved in all stages of atherosclerosis [58]. The inflammatory response in atherosclerosis is regulated by both the innate and adaptive immune systems. The NLRP3 inflammasome, an innate immune-signaling complex, regulates CASP1 activation and the subsequent processing of pro-IL-1β, triggers vascular wall inflammatory responses and leads to the progression of atherosclerosis [59–61]. Many clinical and experimental studies have reported that IL-1β is a proatherogenic cytokine [62–64]. We confirmed the increased expression of TNF, Il-1β, CASP1, and NLRP3 in human complicated atherosclerotic lesions by RNA sequencing. Our hypothesis was that peptides contribute to the progression of atherosclerosis via triggering vascular inflammation. Indeed, exposure of endothelial cells to peptides induced NLRP3 and CASP1, accompanied by the upregulation of TNF, Il-1β, and ICAM1.

Haptoglobin, the primary hemoglobin-binding protein in human plasma, attenuates the adverse biochemical and physiologic effects of extracellular hemoglobin [65]. The inhibitory action of haptoglobin on the peroxide-induced fragmentation of hemoglobin provided might be a further mechanism of its benefit against the pathophysiologic effect of extracellular hemoglobin. Interestingly, under our experimental conditions, haptoglobin failed to bind peptides derived from hemoglobin oxidation.

Our studies demonstrated for the first time that the oxidation of hemoglobin generates peptide fragments and ferryl hemoglobin in complicated atherosclerotic lesions and IVH of the brain. Hemoglobin oxidation triggers dysfunction and induces a proinflammatory response in the endothelium, reflecting its potential pathophysiological role.

## Supplementary information

Supplemental Table
