# Supplementary material for: Hemoglobin oxidation generates globin-derived peptides in atherosclerotic lesions and intraventricular hemorrhage of the brain, provoking endothelial dysfunction
Source: Lab Invest. 2020 Feb 13;100(7):986–1002. doi: 10.1038/s41374-020-0403-x (PMC7311325; doi:10.1038/s41374-020-0403-x)
Supplement: Supplementary file 1 — Supplemental Table [file 41374_2020_403_MOESM1_ESM.pdf]

Atheroma (Ath)

N	%Cov	Accessions	Names	Conf	Sequence	Modifications	Cleavages	dMass	Prec MW	Prec m/z	Theor MW	Theor m/z	Theor z	Sc	Spectrum	Specific	Time	PrecursorSignal	PrecursorElution
1	50,34	sp|P68871|HBB_HUMAN	Hemoglobin subunit beta OS=Homo sapiens GN=HBB PE=1 SV=2	99	EFTPPVQAAYQK			-0,296	1377,397	689,706	1377,693	689,854	2	13	1.1.1.460.4	1	28,1158	0	-1
1	50,34	sp|P68871|HBB_HUMAN	Hemoglobin subunit beta OS=Homo sapiens GN=HBB PE=1 SV=2	99	LLVVYPWTQR			0,132	1273,850	637,932	1273,718	637,866	2	11	1.1.1.518.3	1	33,3199	0	-1
1	50,34	sp|P68871|HBB_HUMAN	Hemoglobin subunit beta OS=Homo sapiens GN=HBB PE=1 SV=2	99	VLGAFSDGLAHLDNLK			0,134	1669,018	557,347	1668,884	557,302	3	14	1.1.1.513.3	1	32,874	0	-1
1	50,34	sp|P68871|HBB_HUMAN	Hemoglobin subunit beta OS=Homo sapiens GN=HBB PE=1 SV=2	99	VVAGVANALAHKYH		missed K-Y@12	-0,092	1448,697	483,906	1448,789	483,937	3	16	1.1.1.451.4	1	27,3973	0	-1
1	50,34	sp|P68871|HBB_HUMAN	Hemoglobin subunit beta OS=Homo sapiens GN=HBB PE=1 SV=2	73,51	KVLGAFSDGLAHLDNLK		missed K-V@1	-0,095	1796,883	599,968	1796,979	600,000	3	11	1.1.1.503.3	1	31,9497	0	-1
1	50,34	sp|P68871|HBB_HUMAN	Hemoglobin subunit beta OS=Homo sapiens GN=HBB PE=1 SV=2	99	EFTPPVQAAYQK			-0,303	1377,390	689,702	1377,693	689,854	2	12	1.1.1.459.4	1	28,0475	0	-1
1	50,34	sp|P68871|HBB_HUMAN	Hemoglobin subunit beta OS=Homo sapiens GN=HBB PE=1 SV=2	99	EFTPPVQAAYQK			-0,272	1377,422	689,718	1377,693	689,854	2	11	1.1.1.458.4	1	27,9793	0	-1
1	50,34	sp|P68871|HBB_HUMAN	Hemoglobin subunit beta OS=Homo sapiens GN=HBB PE=1 SV=2	99	VLGAFSDGLAHLDNLK			0,258	1669,142	557,388	1668,884	557,302	3	14	1.1.1.512.3	1	32,771	0	-1
1	50,34	sp|P68871|HBB_HUMAN	Hemoglobin subunit beta OS=Homo sapiens GN=HBB PE=1 SV=2	99	VVAGVANALAHKYH		missed K-Y@12	-1,389	1447,400	483,474	1448,789	483,937	3	14	1.1.1.453.3	1	27,5338	0	-1
1	50,34	sp|P68871|HBB_HUMAN	Hemoglobin subunit beta OS=Homo sapiens GN=HBB PE=1 SV=2	99	VVAGVANALAHKYH	Deamidated(N)@7	missed K-Y@12	0,336	1450,108	484,377	1449,773	484,265	3	14	1.1.1.454.3	1	27,6368	0	-1
2	58,25	sp|P62805|H4_HUMAN	Histone H4 OS=Homo sapiens GN=HIST1H4A PE=1 SV=2	99	VFLENVIR			0,376	988,946	495,480	988,571	495,293	2	12	1.1.1.496.3	1	31,3693	0	-1
2	58,25	sp|P62805|H4_HUMAN	Histone H4 OS=Homo sapiens GN=HIST1H4A PE=1 SV=2	99	ISGLIYEETR			-0,246	1179,368	590,691	1179,614	590,814	2	9	1.1.1.469.4	1	28,9377	0	-1
2	58,25	sp|P62805|H4_HUMAN	Histone H4 OS=Homo sapiens GN=HIST1H4A PE=1 SV=2	86,86	DAVTYTEHAK			-0,248	1133,287	567,651	1133,535	567,775	2	7	1.1.1.410.4	1	23,591	0	-1
2	58,25	sp|P62805|H4_HUMAN	Histone H4 OS=Homo sapiens GN=HIST1H4A PE=1 SV=2	99	VFLENVIR			-0,151	988,420	495,217	988,571	495,293	2	11	1.1.1.497.4	1	31,4373	0	-1
3	15,85	sp|P00760|TRY1_BOVIN	Cationic trypsin OS=Bos taurus PE=1 SV=3	99	LKSAASLNSR		missed K-S@2	0,210	1045,798	523,907	1045,588	523,801	2	10	1.1.1.407.3	1	23,2475	0	-1
3	15,85	sp|P00760|TRY1_BOVIN	Cationic trypsin OS=Bos taurus PE=1 SV=3	99	SSGTSYPDVLK			0,378	1152,945	577,480	1152,566	577,290	2	11	1.1.1.462.3	1	28,2173	0	-1
3	15,85	sp|P00760|TRY1_BOVIN	Cationic trypsin OS=Bos taurus PE=1 SV=3	71,98	APILSDSSCK	Carbamidomethyl(C)@9		0,229	1076,746	539,380	1076,517	539,266	2	6	1.1.1.428.3	1	25,132	0	-1
3	15,85	sp|P00760|TRY1_BOVIN	Cationic trypsin OS=Bos taurus PE=1 SV=3	59,05	APILSDSSCK	Carbamidomethyl(C)@9		0,245	1076,762	539,388	1076,517	539,266	2	6	1.1.1.429.3	1	25,235	0	-1
3	15,85	sp|P00760|TRY1_BOVIN	Cationic trypsin OS=Bos taurus PE=1 SV=3	88,5	SSGTSYPDVLK			-0,214	1152,353	577,184	1152,566	577,290	2	10	1.1.1.456.4	1	27,8427	0	-1
4	9,019	sp|P62736|ACTA_HUMAN	Actin, aortic smooth muscle OS=Homo sapiens GN=ACTA2 PE=1 SV=1	99	EITALAPSTMK	Oxidation(M)@10		0,202	1176,808	589,411	1176,606	589,310	2	10	1.1.1.442.3	1	26,505	0	-1
4	9,019	sp|P62736|ACTA_HUMAN	Actin, aortic smooth muscle OS=Homo sapiens GN=ACTA2 PE=1 SV=1	91,58	QEYDEAGPSIVHR			0,142	1499,842	500,955	1499,700	500,907	3	12	1.1.1.439.3	1	26,2307	0	-1
4	9,019	sp|P62736|ACTA_HUMAN	Actin, aortic smooth muscle OS=Homo sapiens GN=ACTA2 PE=1 SV=1	60,69	IKIIAPPER		missed K-I@2	0,358	1036,002	519,008	1035,644	518,829	2	7	1.1.1.450.3	1	27,2596	0	-1
4	9,019	sp|P62736|ACTA_HUMAN	Actin, aortic smooth muscle OS=Homo sapiens GN=ACTA2 PE=1 SV=1	57,87	IKIIAPPER		missed K-I@2	-0,164	1035,480	518,747	1035,644	518,829	2	7	1.1.1.449.4	1	27,1913	0	-1
4	9,019	sp|P62736|ACTA_HUMAN	Actin, aortic smooth muscle OS=Homo sapiens GN=ACTA2 PE=1 SV=1	71,79	QEYDEAGPSIVHR			0,269	1499,969	500,997	1499,700	500,907	3	10	1.1.1.438.3	1	26,1278	0	-1
5	19,16	sp|P02662|CASA1_BOVIN	Alpha-S1-casein OS=Bos taurus GN=CSN1S1 PE=1 SV=2	99	FFVAPFPEVFGK			0,116	1383,839	692,927	1383,723	692,869	2	14	1.1.1.548.3	1	36,1002	0	-1
5	19,16	sp|P02662|CASA1_BOVIN	Alpha-S1-casein OS=Bos taurus GN=CSN1S1 PE=1 SV=2	99	YLGYLEQLLR			0,120	1266,818	634,416	1266,697	634,356	2	13	1.1.1.543.3	1	35,6197	0	-1
5	19,16	sp|P02662|CASA1_BOVIN	Alpha-S1-casein OS=Bos taurus GN=CSN1S1 PE=1 SV=2	99	FFVAPFPEVFGK			0,116	1383,839	692,927	1383,723	692,869	2	14	1.1.1.547.3	1	35,9972	0	-1
5	19,16	sp|P02662|CASA1_BOVIN	Alpha-S1-casein OS=Bos taurus GN=CSN1S1 PE=1 SV=2	99	FFVAPFPEVFGK			0,103	1383,825	692,920	1383,723	692,869	2	10	1.1.1.551.3	1	36,4093	0	-1
5	19,16	sp|P02662|CASA1_BOVIN	Alpha-S1-casein OS=Bos taurus GN=CSN1S1 PE=1 SV=2	99	YLGYLEQLLR			0,216	1266,913	634,464	1266,697	634,356	2	11	1.1.1.545.3	1	35,791	0	-1
5	19,16	sp|P02662|CASA1_BOVIN	Alpha-S1-casein OS=Bos taurus GN=CSN1S1 PE=1 SV=2	99	YLGYLEQLLR			0,158	1266,855	634,435	1266,697	634,356	2	13	1.1.1.539.3	1	35,3119	0	-1
6	56,34	sp|P69905|HBA_HUMAN	Hemoglobin subunit alpha OS=Homo sapiens GN=HBA1 PE=1 SV=2	99	TYFPHFDLSHGSAQVK			-0,254	1832,631	611,884	1832,885	611,969	3	15	1.1.1.484.4	1	30,3427	0	-1
6	56,34	sp|P69905|HBA_HUMAN	Hemoglobin subunit alpha OS=Homo sapiens GN=HBA1 PE=1 SV=2	75,34	VGAHAGEYGAEALER			-0,278	1528,449	510,490	1528,727	510,583	3	11	1.1.1.443.4	1	26,6429	0	-1
6	56,34	sp|P69905|HBA_HUMAN	Hemoglobin subunit alpha OS=Homo sapiens GN=HBA1 PE=1 SV=2	61,76	TYFPHFDLSHGSAQVK			-0,207	1832,677	611,900	1832,885	611,969	3	11	1.1.1.483.4	1	30,2745	0	-1
7	8,989	sp|P06703|S10A6_HUMAN	Protein S100-A6 OS=Homo sapiens GN=S100A6 PE=1 SV=1	98,88	LQDAEIAR			0,440	914,922	458,468	914,482	458,248	2	10	1.1.1.420.3	1	24,5169	0	-1
7	8,989	sp|P06703|S10A6_HUMAN	Protein S100-A6 OS=Homo sapiens GN=S100A6 PE=1 SV=1	34,34	LQDAEIAR			0,265	914,747	458,381	914,482	458,248	2	6	1.1.1.427.3	1	25,0293	0	-1
8	2,839	sp|P02649|APOE_HUMAN	Apolipoprotein E OS=Homo sapiens GN=APOE PE=1 SV=1	82,4	LAVYQAGAR			0,273	947,792	474,903	947,519	474,767	2	7	1.1.1.433.3	1	25,6474	0	-1
8	2,839	sp|P02649|APOE_HUMAN	Apolipoprotein E OS=Homo sapiens GN=APOE PE=1 SV=1	75,88	LAVYQAGAR			-0,148	947,371	474,693	947,519	474,767	2	7	1.1.1.435.4	1	25,8533	0	-1

Complicated plaque (Comp)

N	%Cov	Accessions	Names	Conf	Sequence	Modifications	Cleavages	dMass	Prec MW	Prec m/z	Theor MW	Theor m/z	Theor z	Sc	Spectrum	Specific	Time	PrecursorSignal	PrecursorElution
1	67,35	sp|P68871|HBB_HUMAN	Hemoglobin subunit beta OS=Homo sapiens GN=HBB PE=1 SV=2	99	EFTPPVQAAYQK			-0,095	1377,598	689,806	1377,693	689,854	2	14	1.1.1.418.4	1	26,9451	0	-1
1	67,35	sp|P68871|HBB_HUMAN	Hemoglobin subunit beta OS=Homo sapiens GN=HBB PE=1 SV=2	99	LLVVYPWTQR			-0,036	1273,682	637,848	1273,718	637,866	2	13	1.1.1.483.4	1	32,3251	0	-1
1	67,35	sp|P68871|HBB_HUMAN	Hemoglobin subunit beta OS=Homo sapiens GN=HBB PE=1 SV=2	99	VLGAFSDGLAHLDNLK			0,507	1669,391	557,471	1668,884	557,302	3	14	1.1.1.479.3	1	31,9827	0	-1
1	67,35	sp|P68871|HBB_HUMAN	Hemoglobin subunit beta OS=Homo sapiens GN=HBB PE=1 SV=2	96,15	VVAGVANALAHKYH		missed K-Y@12	-1,892	1446,897	483,306	1448,789	483,937	3	12	1.1.1.417.3	1	26,842	0	-1
1	67,35	sp|P68871|HBB_HUMAN	Hemoglobin subunit beta OS=Homo sapiens GN=HBB PE=1 SV=2	98,84	VLGAFSDGLAHLDNLK			0,484	1669,367	557,463	1668,884	557,302	3	13	1.1.1.481.3	1	32,1539	0	-1
1	67,35	sp|P68871|HBB_HUMAN	Hemoglobin subunit beta OS=Homo sapiens GN=HBB PE=1 SV=2	96,44	VLGAFSDGLAHLDNLK			0,129	1669,012	557,345	1668,884	557,302	3	13	1.1.1.477.4	1	31,8116	0	-1
1	67,35	sp|P68871|HBB_HUMAN	Hemoglobin subunit beta OS=Homo sapiens GN=HBB PE=1 SV=2	89,76	VLGAFSDGLAHLDNLK			-0,073	1668,810	557,277	1668,884	557,302	3	11	1.1.1.485.4	1	32,4611	0	-1
1	67,35	sp|P68871|HBB_HUMAN	Hemoglobin subunit beta OS=Homo sapiens GN=HBB PE=1 SV=2	42,59	VVAGVANALAHKYH		missed K-Y@12	-0,331	1448,458	483,827	1448,789	483,937	3	9	1.1.1.434.3	1	28,4206	0	-1
2	56,31	sp|P62805|H4_HUMAN	Histone H4 OS=Homo sapiens GN=HIST1H4A PE=1 SV=2	99	ISGLIYEETR			0,438	1180,052	591,033	1179,614	590,814	2	12	1.1.1.430.3	1	28,0779	0	-1
2	56,31	sp|P62805|H4_HUMAN	Histone H4 OS=Homo sapiens GN=HIST1H4A PE=1 SV=2	95,78	TVTAMDVVYALKR		missed K-R@12	-0,345	1465,451	489,491	1465,796	489,606	3	10	1.1.1.476.3	1	31,7085	0	-1
2	56,31	sp|P62805|H4_HUMAN	Histone H4 OS=Homo sapiens GN=HIST1H4A PE=1 SV=2	76,94	DNIQGITKPAIR			-0,128	1324,619	663,317	1324,746	663,380	2	7	1.1.1.406.4	1	25,9522	0	-1
2	56,31	sp|P62805|H4_HUMAN	Histone H4 OS=Homo sapiens GN=HIST1H4A PE=1 SV=2	60,79	DNIQGITKPAIR			0,121	1324,868	663,441	1324,746	663,380	2	8	1.1.1.405.3	1	25,8492	0	-1
2	56,31	sp|P62805|H4_HUMAN	Histone H4 OS=Homo sapiens GN=HIST1H4A PE=1 SV=2	61,51	TVTAMDVVYALKR	Oxidation(M)@5	missed K-R@12	-0,050	1481,741	494,921	1481,791	494,938	3	10	1.1.1.451.4	1	29,6878	0	-1
3	15,85	sp|P00760|TRY1_BOVIN	Cationic trypsin OS=Bos taurus PE=1 SV=3	99	LKSAASLNSR		missed K-S@2	0,336	1045,924	523,970	1045,588	523,801	2	9	1.1.1.368.3	1	22,2818	0	-1
3	15,85	sp|P00760|TRY1_BOVIN	Cationic trypsin OS=Bos taurus PE=1 SV=3	99	SSGTSYPDVLK			-0,015	1152,551	577,283	1152,566	577,290	2	15	1.1.1.419.4	1	27,0133	0	-1
3	15,85	sp|P00760|TRY1_BOVIN	Cationic trypsin OS=Bos taurus PE=1 SV=3	33,47	APILSDSSCK	Carbamidomethyl(C)@9		-0,112	1076,406	539,210	1076,517	539,266	2	6	1.1.1.388.4	1	24,2364	0	-1
3	15,85	sp|P00760|TRY1_BOVIN	Cationic trypsin OS=Bos taurus PE=1 SV=3	87,69	LKSAASLNSR		missed K-S@2	0,314	1045,902	523,958	1045,588	523,801	2	8	1.1.1.367.3	1	22,1789	0	-1
3	15,85	sp|P00760|TRY1_BOVIN	Cationic trypsin OS=Bos taurus PE=1 SV=3	44,1	LKSAASLNSR		missed K-S@2	0,305	1045,893	523,954	1045,588	523,801	2	6	1.1.1.369.3	1	22,3844	0	-1
4	19,16	sp|P02662|CASA1_BOVIN	Alpha-S1-casein OS=Bos taurus GN=CSN1S1 PE=1 SV=2	99	FFVAPFPEVFGK			0,305	1384,027	693,021	1383,723	692,869	2	13	1.1.1.523.3	1	35,5349	0	-1
4	19,16	sp|P02662|CASA1_BOVIN	Alpha-S1-casein OS=Bos taurus GN=CSN1S1 PE=1 SV=2	99	YLGYLEQLLR			1,287	1267,984	634,999	1266,697	634,356	2	14	1.1.1.516.3	1	34,8503	0	-1
4	19,16	sp|P02662|CASA1_BOVIN	Alpha-S1-casein OS=Bos taurus GN=CSN1S1 PE=1 SV=2	99	FFVAPFPEVFGK			-0,749	1382,974	692,494	1383,723	692,869	2	12	1.1.1.522.3	1	35,4321	0	-1
4	19,16	sp|P02662|CASA1_BOVIN	Alpha-S1-casein OS=Bos taurus GN=CSN1S1 PE=1 SV=2	99	FFVAPFPEVFGK			-0,829	1382,893	692,454	1383,723	692,869	2	12	1.1.1.524.3	1	35,6378	0	-1
4	19,16	sp|P02662|CASA1_BOVIN	Alpha-S1-casein OS=Bos taurus GN=CSN1S1 PE=1 SV=2	99	FFVAPFPEVFGK			0,128	1383,851	692,933	1383,723	692,869	2	14	1.1.1.521.3	1	35,3293	0	-1
4	19,16	sp|P02662|CASA1_BOVIN	Alpha-S1-casein OS=Bos taurus GN=CSN1S1 PE=1 SV=2	99	YLGYLEQLLR			1,321	1268,018	635,016	1266,697	634,356	2	16	1.1.1.517.3	1	34,9531	0	-1
4	19,16	sp|P02662|CASA1_BOVIN	Alpha-S1-casein OS=Bos taurus GN=CSN1S1 PE=1 SV=2	99	YLGYLEQLLR			-0,090	1266,607	634,311	1266,697	634,356	2	12	1.1.1.521.4	1	35,3641	0	-1
4	19,16	sp|P02662|CASA1_BOVIN	Alpha-S1-casein OS=Bos taurus GN=CSN1S1 PE=1 SV=2	99	YLGYLEQLLR			0,337	1267,034	634,524	1266,697	634,356	2	15	1.1.1.515.3	1	34,7475	0	-1
4	19,16	sp|P02662|CASA1_BOVIN	Alpha-S1-casein OS=Bos taurus GN=CSN1S1 PE=1 SV=2	99	YLGYLEQLLR			0,310	1267,008	634,511	1266,697	634,356	2	14	1.1.1.518.3	1	35,0557	0	-1
5	42,25	sp|P69905|HBA_HUMAN	Hemoglobin subunit alpha OS=Homo sapiens GN=HBA1 PE=1 SV=2	98,88	VGAHAGEYGAEALER			-0,080	1528,647	510,556	1528,727	510,583	3	13	1.1.1.401.4	1	25,4717	0	-1
5	42,25	sp|P69905|HBA_HUMAN	Hemoglobin subunit alpha OS=Homo sapiens GN=HBA1 PE=1 SV=2	85,86	MFLSFPTTK	Oxidation(M)@1		0,054	1086,595	544,305	1086,542	544,278	2	8	1.1.1.463.4	1	30,7162	0	-1
5	42,25	sp|P69905|HBA_HUMAN	Hemoglobin subunit alpha OS=Homo sapiens GN=HBA1 PE=1 SV=2	75,51	TYFPHFDLSHGSAQVK			0,035	1832,919	611,980	1832,885	611,969	3	12	1.1.1.447.4	1	29,4143	0	-1
5	42,25	sp|P69905|HBA_HUMAN	Hemoglobin subunit alpha OS=Homo sapiens GN=HBA1 PE=1 SV=2	41,64	VLSPADKTNVK		cleaved M-V@N-term; missed K-T@7	0,266	1170,927	586,471	1170,661	586,338	2	7	1.1.1.372.3	1	22,6583	0	-1
5	42,25	sp|P69905|HBA_HUMAN	Hemoglobin subunit alpha OS=Homo sapiens GN=HBA1 PE=1 SV=2	38,44	MFLSFPTTK	Oxidation(M)@1		0,004	1086,546	544,280	1086,542	544,278	2	7	1.1.1.461.4	1	30,5451	0	-1
5	42,25	sp|P69905|HBA_HUMAN	Hemoglobin subunit alpha OS=Homo sapiens GN=HBA1 PE=1 SV=2	92,76	VGAHAGEYGAEALER			-0,563	1528,164	510,395	1528,727	510,583	3	14	1.1.1.402.3	1	25,5401	0	-1
6	7,389	sp|P02768|ALBU_HUMAN	Serum albumin OS=Homo sapiens GN=ALB PE=1 SV=2	99	LVNEVTEFAK			-0,114	1148,494	575,254	1148,608	575,311	2	10	1.1.1.429.4	1	28,0095	0	-1
7	19,1	sp|P06703|S10A6_HUMAN	Protein S100-A6 OS=Homo sapiens GN=S100A6 PE=1 SV=1	98,92	LQDAEIAR			0,543	915,025	458,520	914,482	458,248	2	12	1.1.1.383.3	1	23,7216	0	-1
9	11,99	sp|P02649|APOE_HUMAN	Apolipoprotein E OS=Homo sapiens GN=APOE PE=1 SV=1	98,76	LAVYQAGAR			-0,118	947,401	474,708	947,519	474,767	2	10	1.1.1.394.4	1	24,785	0	-1

Late phase of intraventrical hemorrhage (LIVH)

<table>
<tr><th>N</th><th>%Cov</th><th>Accessions</th><th>Names</th><th>Conf</th><th>Sequence</th><th>Modifications</th><th>Cleavages</th><th>dMass</th><th>Prec MW</th><th>Prec m/z</th><th>Theor MW</th><th>Theor m/z</th><th>Theor z</th><th>Sc</th><th>Spectrum</th><th>Specific</th><th>Time</th><th>PrecursorSignal</th><th>PrecursorElution</th></tr>
<tr><td>1</td><td>71,13</td><td>sp|P69905|HBA_HUMAN</td><td>Hemoglobin subunit alpha OS=Homo sapiens GN=HBA1 PE=1 SV=2</td><td>99</td><td>FLASVSTVLTSK</td><td></td><td></td><td>-0,122</td><td>1251,585</td><td>626,800</td><td>1251,707</td><td>626,861</td><td>2</td><td>13</td><td>1.1.1.442.4</td><td>1</td><td>30,4065</td><td>0</td><td>-1</td></tr>
<tr><td>1</td><td>71,13</td><td>sp|P69905|HBA_HUMAN</td><td>Hemoglobin subunit alpha OS=Homo sapiens GN=HBA1 PE=1 SV=2</td><td>99</td><td>MFLSFPTTK</td><td>Oxidation(M)@1</td><td></td><td>1,172</td><td>1087,714</td><td>544,864</td><td>1086,542</td><td>544,278</td><td>2</td><td>12</td><td>1.1.1.440.3</td><td>1</td><td>30,1657</td><td>0</td><td>-1</td></tr>
<tr><td>1</td><td>71,13</td><td>sp|P69905|HBA_HUMAN</td><td>Hemoglobin subunit alpha OS=Homo sapiens GN=HBA1 PE=1 SV=2</td><td>99</td><td>TYFPHFDLSHGSAQVK</td><td></td><td></td><td>-0,090</td><td>1832,794</td><td>611,939</td><td>1832,885</td><td>611,969</td><td>3</td><td>16</td><td>1.1.1.430.3</td><td>1</td><td>29,2747</td><td>0</td><td>-1</td></tr>
<tr><td>1</td><td>71,13</td><td>sp|P69905|HBA_HUMAN</td><td>Hemoglobin subunit alpha OS=Homo sapiens GN=HBA1 PE=1 SV=2</td><td>99</td><td>VGAHAGEYGAEALER</td><td></td><td></td><td>-0,313</td><td>1528,414</td><td>510,479</td><td>1528,727</td><td>510,583</td><td>3</td><td>16</td><td>1.1.1.387.4</td><td>1</td><td>25,3416</td><td>0</td><td>-1</td></tr>
<tr><td>1</td><td>71,13</td><td>sp|P69905|HBA_HUMAN</td><td>Hemoglobin subunit alpha OS=Homo sapiens GN=HBA1 PE=1 SV=2</td><td>68,11</td><td>ALSALSDLHAHKLR</td><td></td><td>cleaved N-A@N-term; missed K-L@12</td><td>-0,497</td><td>1530,366</td><td>511,129</td><td>1530,863</td><td>511,295</td><td>3</td><td>11</td><td>1.1.1.404.4</td><td>1</td><td>26,6665</td><td>0</td><td>-1</td></tr>
<tr><td>1</td><td>71,13</td><td>sp|P69905|HBA_HUMAN</td><td>Hemoglobin subunit alpha OS=Homo sapiens GN=HBA1 PE=1 SV=2</td><td>99</td><td>FLASVSTVLTSK</td><td></td><td></td><td>-0,197</td><td>1251,510</td><td>626,762</td><td>1251,707</td><td>626,861</td><td>2</td><td>13</td><td>1.1.1.441.4</td><td>1</td><td>30,3035</td><td>0</td><td>-1</td></tr>
<tr><td>1</td><td>71,13</td><td>sp|P69905|HBA_HUMAN</td><td>Hemoglobin subunit alpha OS=Homo sapiens GN=HBA1 PE=1 SV=2</td><td>99</td><td>MFLSFPTTK</td><td>Oxidation(M)@1</td><td></td><td>0,181</td><td>1086,724</td><td>544,369</td><td>1086,542</td><td>544,278</td><td>2</td><td>11</td><td>1.1.1.442.3</td><td>1</td><td>30,3717</td><td>0</td><td>-1</td></tr>
<tr><td>1</td><td>71,13</td><td>sp|P69905|HBA_HUMAN</td><td>Hemoglobin subunit alpha OS=Homo sapiens GN=HBA1 PE=1 SV=2</td><td>99</td><td>MFLSFPTTK</td><td>Oxidation(M)@1</td><td></td><td>0,223</td><td>1086,766</td><td>544,390</td><td>1086,542</td><td>544,278</td><td>2</td><td>11</td><td>1.1.1.441.3</td><td>1</td><td>30,2687</td><td>0</td><td>-1</td></tr>
<tr><td>1</td><td>71,13</td><td>sp|P69905|HBA_HUMAN</td><td>Hemoglobin subunit alpha OS=Homo sapiens GN=HBA1 PE=1 SV=2</td><td>99</td><td>MFLSFPTTK</td><td>Oxidation(M)@1</td><td></td><td>-0,248</td><td>1086,294</td><td>544,155</td><td>1086,542</td><td>544,278</td><td>2</td><td>9</td><td>1.1.1.445.4</td><td>1</td><td>30,6808</td><td>0</td><td>-1</td></tr>
<tr><td>1</td><td>71,13</td><td>sp|P69905|HBA_HUMAN</td><td>Hemoglobin subunit alpha OS=Homo sapiens GN=HBA1 PE=1 SV=2</td><td>76,94</td><td>MFLSFPTTK</td><td>Oxidation(M)@1</td><td></td><td>-0,179</td><td>1086,363</td><td>544,189</td><td>1086,542</td><td>544,278</td><td>2</td><td>7</td><td>1.1.1.450.4</td><td>1</td><td>31,1956</td><td>0</td><td>-1</td></tr>
<tr><td>1</td><td>71,13</td><td>sp|P69905|HBA_HUMAN</td><td>Hemoglobin subunit alpha OS=Homo sapiens GN=HBA1 PE=1 SV=2</td><td>21,54</td><td>MFLSFPTTK</td><td>Oxidation(M)@1</td><td></td><td>-0,202</td><td>1086,340</td><td>544,177</td><td>1086,542</td><td>544,278</td><td>2</td><td>6</td><td>1.1.1.470.4</td><td>1</td><td>33,045</td><td>0</td><td>-1</td></tr>
<tr><td>1</td><td>71,13</td><td>sp|P69905|HBA_HUMAN</td><td>Hemoglobin subunit alpha OS=Homo sapiens GN=HBA1 PE=1 SV=2</td><td>17,28</td><td>MFLSFPTTK</td><td>Oxidation(M)@1</td><td></td><td>-0,408</td><td>1086,134</td><td>544,074</td><td>1086,542</td><td>544,278</td><td>2</td><td>9</td><td>1.1.1.439.3</td><td>1</td><td>30,0627</td><td>0</td><td>-1</td></tr>
<tr><td>1</td><td>71,13</td><td>sp|P69905|HBA_HUMAN</td><td>Hemoglobin subunit alpha OS=Homo sapiens GN=HBA1 PE=1 SV=2</td><td>99</td><td>TYFPHFDLSHGSAQVK</td><td></td><td></td><td>-0,141</td><td>1832,744</td><td>611,922</td><td>1832,885</td><td>611,969</td><td>3</td><td>14</td><td>1.1.1.431.3</td><td>1</td><td>29,3429</td><td>0</td><td>-1</td></tr>
<tr><td>1</td><td>71,13</td><td>sp|P69905|HBA_HUMAN</td><td>Hemoglobin subunit alpha OS=Homo sapiens GN=HBA1 PE=1 SV=2</td><td>30,8</td><td>TYFPHFDLSHGSAQVK</td><td></td><td></td><td>-0,251</td><td>1832,634</td><td>611,885</td><td>1832,885</td><td>611,969</td><td>3</td><td>11</td><td>1.1.1.433.4</td><td>1</td><td>29,549</td><td>0</td><td>-1</td></tr>
<tr><td>1</td><td>71,13</td><td>sp|P69905|HBA_HUMAN</td><td>Hemoglobin subunit alpha OS=Homo sapiens GN=HBA1 PE=1 SV=2</td><td>99</td><td>VGAHAGEYGAEALER</td><td></td><td></td><td>-0,332</td><td>1528,395</td><td>510,472</td><td>1528,727</td><td>510,583</td><td>3</td><td>15</td><td>1.1.1.389.4</td><td>1</td><td>25,4769</td><td>0</td><td>-1</td></tr>
<tr><td>1</td><td>71,13</td><td>sp|P69905|HBA_HUMAN</td><td>Hemoglobin subunit alpha OS=Homo sapiens GN=HBA1 PE=1 SV=2</td><td>99</td><td>VGAHAGEYGAEALER</td><td></td><td></td><td>-0,405</td><td>1528,322</td><td>510,448</td><td>1528,727</td><td>510,583</td><td>3</td><td>13</td><td>1.1.1.386.4</td><td>1</td><td>25,2391</td><td>0</td><td>-1</td></tr>
<tr><td></td><td></td><td></td><td></td><td></td><td></td><td></td><td></td><td></td><td></td><td></td><td></td><td></td><td></td><td></td><td></td><td></td><td></td><td></td><td></td></tr>
<tr><td>2</td><td>38,78</td><td>sp|P69891|HBG1_HUMAN</td><td>Hemoglobin subunit gamma-1 OS=Homo sapiens GN=HBG1 PE=1 SV=2</td><td>99</td><td>KVLTSLGDAIK</td><td></td><td>missed K-V@1</td><td>-0,228</td><td>1143,459</td><td>572,737</td><td>1143,686</td><td>572,850</td><td>2</td><td>12</td><td>1.1.1.410.4</td><td>0</td><td>27,2833</td><td>0</td><td>-1</td></tr>
<tr><td>2</td><td>38,78</td><td>sp|P69891|HBG1_HUMAN</td><td>Hemoglobin subunit gamma-1 OS=Homo sapiens GN=HBG1 PE=1 SV=2</td><td>99</td><td>LLGNVLVTVLAIHFGK</td><td></td><td></td><td>1,348</td><td>1694,377</td><td>565,800</td><td>1693,029</td><td>565,350</td><td>3</td><td>13</td><td>1.1.1.527.3</td><td>0</td><td>38,4852</td><td>0</td><td>-1</td></tr>
<tr><td>2</td><td>38,78</td><td>sp|P69891|HBG1_HUMAN</td><td>Hemoglobin subunit gamma-1 OS=Homo sapiens GN=HBG1 PE=1 SV=2</td><td>99</td><td>LLVVYPWTQR</td><td></td><td></td><td>0,062</td><td>1273,780</td><td>637,897</td><td>1273,718</td><td>637,866</td><td>2</td><td>14</td><td>1.1.1.465.3</td><td>0</td><td>32,4961</td><td>0</td><td>-1</td></tr>
<tr><td>2</td><td>38,78</td><td>sp|P69891|HBG1_HUMAN</td><td>Hemoglobin subunit gamma-1 OS=Homo sapiens GN=HBG1 PE=1 SV=2</td><td>99</td><td>MVTAVASALSSR</td><td>Oxidation(M)@1</td><td></td><td>-0,197</td><td>1207,427</td><td>604,721</td><td>1207,623</td><td>604,819</td><td>2</td><td>19</td><td>1.1.1.402.4</td><td>1</td><td>26,4961</td><td>0</td><td>-1</td></tr>
<tr><td>2</td><td>38,78</td><td>sp|P69891|HBG1_HUMAN</td><td>Hemoglobin subunit gamma-1 OS=Homo sapiens GN=HBG1 PE=1 SV=2</td><td>99</td><td>VLTSLGDAIK</td><td></td><td></td><td>0,906</td><td>1016,497</td><td>509,256</td><td>1015,591</td><td>508,803</td><td>2</td><td>13</td><td>1.1.1.418.3</td><td>0</td><td>28,0725</td><td>0</td><td>-1</td></tr>
<tr><td>2</td><td>38,78</td><td>sp|P69891|HBG1_HUMAN</td><td>Hemoglobin subunit gamma-1 OS=Homo sapiens GN=HBG1 PE=1 SV=2</td><td>71,91</td><td>LLGNVLVTVLAIHFGK</td><td></td><td></td><td>0,483</td><td>1693,512</td><td>565,511</td><td>1693,029</td><td>565,350</td><td>3</td><td>11</td><td>1.1.1.526.3</td><td>0</td><td>38,3824</td><td>0</td><td>-1</td></tr>
<tr><td>2</td><td>38,78</td><td>sp|P69891|HBG1_HUMAN</td><td>Hemoglobin subunit gamma-1 OS=Homo sapiens GN=HBG1 PE=1 SV=2</td><td>46,16</td><td>LLGNVLVTVLAIHFGK</td><td></td><td></td><td>0,302</td><td>1693,331</td><td>565,451</td><td>1693,029</td><td>565,350</td><td>3</td><td>9</td><td>1.1.1.530.3</td><td>0</td><td>38,7587</td><td>0</td><td>-1</td></tr>
<tr><td>2</td><td>38,78</td><td>sp|P69891|HBG1_HUMAN</td><td>Hemoglobin subunit gamma-1 OS=Homo sapiens GN=HBG1 PE=1 SV=2</td><td>99</td><td>LLVVYPWTQR</td><td></td><td></td><td>0,113</td><td>1273,832</td><td>637,923</td><td>1273,718</td><td>637,866</td><td>2</td><td>14</td><td>1.1.1.464.3</td><td>0</td><td>32,3932</td><td>0</td><td>-1</td></tr>
<tr><td>2</td><td>38,78</td><td>sp|P69891|HBG1_HUMAN</td><td>Hemoglobin subunit gamma-1 OS=Homo sapiens GN=HBG1 PE=1 SV=2</td><td>99</td><td>LLVVYPWTQR</td><td></td><td></td><td>0,089</td><td>1273,807</td><td>637,911</td><td>1273,718</td><td>637,866</td><td>2</td><td>14</td><td>1.1.1.463.3</td><td>0</td><td>32,2904</td><td>0</td><td>-1</td></tr>
<tr><td>2</td><td>38,78</td><td>sp|P69891|HBG1_HUMAN</td><td>Hemoglobin subunit gamma-1 OS=Homo sapiens GN=HBG1 PE=1 SV=2</td><td>99</td><td>LLVVYPWTQR</td><td></td><td></td><td>-0,036</td><td>1273,682</td><td>637,848</td><td>1273,718</td><td>637,866</td><td>2</td><td>13</td><td>1.1.1.462.3</td><td>0</td><td>32,1875</td><td>0</td><td>-1</td></tr>
<tr><td>2</td><td>38,78</td><td>sp|P69891|HBG1_HUMAN</td><td>Hemoglobin subunit gamma-1 OS=Homo sapiens GN=HBG1 PE=1 SV=2</td><td>99</td><td>LLVVYPWTQR</td><td></td><td></td><td>0,164</td><td>1273,883</td><td>637,949</td><td>1273,718</td><td>637,866</td><td>2</td><td>12</td><td>1.1.1.468.3</td><td>0</td><td>32,8044</td><td>0</td><td>-1</td></tr>
<tr><td>2</td><td>38,78</td><td>sp|P69891|HBG1_HUMAN</td><td>Hemoglobin subunit gamma-1 OS=Homo sapiens GN=HBG1 PE=1 SV=2</td><td>99</td><td>LLVVYPWTQR</td><td></td><td></td><td>0,089</td><td>1273,807</td><td>637,911</td><td>1273,718</td><td>637,866</td><td>2</td><td>11</td><td>1.1.1.466.3</td><td>0</td><td>32,599</td><td>0</td><td>-1</td></tr>
<tr><td>2</td><td>38,78</td><td>sp|P69891|HBG1_HUMAN</td><td>Hemoglobin subunit gamma-1 OS=Homo sapiens GN=HBG1 PE=1 SV=2</td><td>99</td><td>LLVVYPWTQR</td><td></td><td></td><td>0,169</td><td>1273,887</td><td>637,951</td><td>1273,718</td><td>637,866</td><td>2</td><td>10</td><td>1.1.1.471.3</td><td>0</td><td>33,113</td><td>0</td><td>-1</td></tr>
<tr><td>2</td><td>38,78</td><td>sp|P69891|HBG1_HUMAN</td><td>Hemoglobin subunit gamma-1 OS=Homo sapiens GN=HBG1 PE=1 SV=2</td><td>98,28</td><td>LLVVYPWTQR</td><td></td><td></td><td>0,222</td><td>1273,941</td><td>637,978</td><td>1273,718</td><td>637,866</td><td>2</td><td>9</td><td>1.1.1.469.3</td><td>0</td><td>32,9072</td><td>0</td><td>-1</td></tr>
<tr><td>2</td><td>38,78</td><td>sp|P69891|HBG1_HUMAN</td><td>Hemoglobin subunit gamma-1 OS=Homo sapiens GN=HBG1 PE=1 SV=2</td><td>22,28</td><td>MVTAVASALSSRYH</td><td>Oxidation(M)@1</td><td>missed R-Y@12</td><td>-0,499</td><td>1507,246</td><td>503,423</td><td>1507,745</td><td>503,589</td><td>3</td><td>10</td><td>1.1.1.408.3</td><td>1</td><td>27,0426</td><td>0</td><td>-1</td></tr>
<tr><td>2</td><td>38,78</td><td>sp|P69891|HBG1_HUMAN</td><td>Hemoglobin subunit gamma-1 OS=Homo sapiens GN=HBG1 PE=1 SV=2</td><td>90,1</td><td>VLTSLGDAIK</td><td></td><td></td><td>-0,395</td><td>1015,196</td><td>508,605</td><td>1015,591</td><td>508,803</td><td>2</td><td>11</td><td>1.1.1.419.3</td><td>0</td><td>28,1757</td><td>0</td><td>-1</td></tr>
<tr><td>2</td><td>38,78</td><td>sp|P69891|HBG1_HUMAN</td><td>Hemoglobin subunit gamma-1 OS=Homo sapiens GN=HBG1 PE=1 SV=2</td><td>84,56</td><td>VLTSLGDAIK</td><td></td><td></td><td>0,212</td><td>1015,804</td><td>508,909</td><td>1015,591</td><td>508,803</td><td>2</td><td>11</td><td>1.1.1.421.3</td><td>0</td><td>28,3815</td><td>0</td><td>-1</td></tr>
<tr><td>2</td><td>38,78</td><td>sp|P69891|HBG1_HUMAN</td><td>Hemoglobin subunit gamma-1 OS=Homo sapiens GN=HBG1 PE=1 SV=2</td><td>35,8</td><td>VLTSLGDAIK</td><td></td><td></td><td>-0,319</td><td>1015,272</td><td>508,644</td><td>1015,591</td><td>508,803</td><td>2</td><td>8</td><td>1.1.1.422.4</td><td>0</td><td>28,5194</td><td>0</td><td>-1</td></tr>
<tr><td></td><td></td><td></td><td></td><td></td><td></td><td></td><td></td><td></td><td></td><td></td><td></td><td></td><td></td><td></td><td></td><td></td><td></td><td></td><td></td></tr>
<tr><td>3</td><td>62,01</td><td>sp|P0CG48|UBC_HUMAN</td><td>Polyubiquitin-C OS=Homo sapiens GN=UBC PE=1 SV=3</td><td>99</td><td>TITLEVEPSDTIENVK</td><td></td><td></td><td>-0,054</td><td>1786,866</td><td>894,440</td><td>1786,920</td><td>894,467</td><td>2</td><td>17</td><td>1.1.1.432.4</td><td>1</td><td>29,4459</td><td>0</td><td>-1</td></tr>
<tr><td>3</td><td>62,01</td><td>sp|P0CG48|UBC_HUMAN</td><td>Polyubiquitin-C OS=Homo sapiens GN=UBC PE=1 SV=3</td><td>99</td><td>TLSDYNIQKESTLHLVLR</td><td></td><td>missed K-E@9</td><td>-0,241</td><td>2128,907</td><td>710,643</td><td>2129,148</td><td>710,723</td><td>3</td><td>15</td><td>1.1.1.437.4</td><td>1</td><td>29,8915</td><td>0</td><td>-1</td></tr>
<tr><td>3</td><td>62,01</td><td>sp|P0CG48|UBC_HUMAN</td><td>Polyubiquitin-C OS=Homo sapiens GN=UBC PE=1 SV=3</td><td>98,44</td><td>IQDKEGIPPDQQR</td><td></td><td>missed K-E@4</td><td>-0,570</td><td>1522,204</td><td>508,409</td><td>1522,774</td><td>508,599</td><td>3</td><td>11</td><td>1.1.1.365.4</td><td>1</td><td>23,3332</td><td>0</td><td>-1</td></tr>
<tr><td>3</td><td>62,01</td><td>sp|P0CG48|UBC_HUMAN</td><td>Polyubiquitin-C OS=Homo sapiens GN=UBC PE=1 SV=3</td><td>24,6</td><td>ESTLHLVLR</td><td></td><td></td><td>-0,251</td><td>1066,363</td><td>534,189</td><td>1066,614</td><td>534,314</td><td>2</td><td>6</td><td>1.1.1.423.4</td><td>1</td><td>28,6226</td><td>0</td><td>-1</td></tr>
<tr><td>3</td><td>62,01</td><td>sp|P0CG48|UBC_HUMAN</td><td>Polyubiquitin-C OS=Homo sapiens GN=UBC PE=1 SV=3</td><td>95,88</td><td>IQDKEGIPPDQQR</td><td></td><td>missed K-E@4</td><td>-0,485</td><td>1522,289</td><td>508,437</td><td>1522,774</td><td>508,599</td><td>3</td><td>11</td><td>1.1.1.378.4</td><td>1</td><td>24,5941</td><td>0</td><td>-1</td></tr>
<tr><td>3</td><td>62,01</td><td>sp|P0CG48|UBC_HUMAN</td><td>Polyubiquitin-C OS=Homo sapiens GN=UBC PE=1 SV=3</td><td>93,91</td><td>IQDKEGIPPDQQR</td><td></td><td>missed K-E@4</td><td>-0,438</td><td>1522,336</td><td>508,453</td><td>1522,774</td><td>508,599</td><td>3</td><td>11</td><td>1.1.1.358.4</td><td>1</td><td>22,6519</td><td>0</td><td>-1</td></tr>
<tr><td>3</td><td>62,01</td><td>sp|P0CG48|UBC_HUMAN</td><td>Polyubiquitin-C OS=Homo sapiens GN=UBC PE=1 SV=3</td><td>59,05</td><td>IQDKEGIPPDQQR</td><td></td><td>missed K-E@4</td><td>0,205</td><td>1522,979</td><td>508,667</td><td>1522,774</td><td>508,599</td><td>3</td><td>9</td><td>1.1.1.377.3</td><td>1</td><td>24,4569</td><td>0</td><td>-1</td></tr>
<tr><td>3</td><td>62,01</td><td>sp|P0CG48|UBC_HUMAN</td><td>Polyubiquitin-C OS=Homo sapiens GN=UBC PE=1 SV=3</td><td>31,65</td><td>IQDKQGIPPDQQR</td><td>Deamidated(Q)@5</td><td>missed K-Q@4</td><td>-0,570</td><td>1522,204</td><td>508,409</td><td>1522,774</td><td>508,599</td><td>3</td><td>11</td><td>1.1.1.365.4</td><td>1</td><td>23,3332</td><td>0</td><td>-1</td></tr>
<tr><td>3</td><td>62,01</td><td>sp|P0CG48|UBC_HUMAN</td><td>Polyubiquitin-C OS=Homo sapiens GN=UBC PE=1 SV=3</td><td>31,45</td><td>IQDKQGIPPDQQR</td><td>Deamidated(Q)@5</td><td>missed K-Q@4</td><td>-0,485</td><td>1522,289</td><td>508,437</td><td>1522,774</td><td>508,599</td><td>3</td><td>11</td><td>1.1.1.378.4</td><td>1</td><td>24,5941</td><td>0</td><td>-1</td></tr>
<tr><td>3</td><td>62,01</td><td>sp|P0CG48|UBC_HUMAN</td><td>Polyubiquitin-C OS=Homo sapiens GN=UBC PE=1 SV=3</td><td>30,96</td><td>IQDKQGIPPDQQR</td><td>Deamidated(Q)@5</td><td>missed K-Q@4</td><td>-0,438</td><td>1522,336</td><td>508,453</td><td>1522,774</td><td>508,599</td><td>3</td><td>11</td><td>1.1.1.358.4</td><td>1</td><td>22,6519</td><td>0</td><td>-1</td></tr>
<tr><td>3</td><td>62,01</td><td>sp|P0CG48|UBC_HUMAN</td><td>Polyubiquitin-C OS=Homo sapiens GN=UBC PE=1 SV=3</td><td>99</td><td>TLSDYNIQKESTLHLVLR</td><td></td><td>missed K-E@9</td><td>-0,247</td><td>2128,901</td><td>710,641</td><td>2129,148</td><td>710,723</td><td>3</td><td>13</td><td>1.1.1.435.4</td><td>1</td><td>29,7203</td><td>0</td><td>-1</td></tr>
<tr><td>3</td><td>62,01</td><td>sp|P0CG48|UBC_HUMAN</td><td>Polyubiquitin-C OS=Homo sapiens GN=UBC PE=1 SV=3</td><td>18,31</td><td>TLSDYNIQKESTLHLVLR</td><td></td><td>missed K-E@9</td><td>-0,241</td><td>2128,907</td><td>710,643</td><td>2129,148</td><td>710,723</td><td>3</td><td>10</td><td>1.1.1.438.4</td><td>1</td><td>29,9945</td><td>0</td><td>-1</td></tr>
<tr><td></td><td></td><td></td><td></td><td></td><td></td><td></td><td></td><td></td><td></td><td></td><td></td><td></td><td></td><td></td><td></td><td></td><td></td><td></td><td></td></tr>
<tr><td>4</td><td>28,97</td><td>sp|P02662|CASA1_BOVIN</td><td>Alpha-S1-casein OS=Bos taurus GN=CSN1S1 PE=1 SV=2</td><td>99</td><td>FFVAPFPEVFGK</td><td></td><td></td><td>0,103</td><td>1383,826</td><td>692,920</td><td>1383,723</td><td>692,869</td><td>2</td><td>12</td><td>1.1.1.495.3</td><td>1</td><td>35,3014</td><td>0</td><td>-1</td></tr>
<tr><td>4</td><td>28,97</td><td>sp|P02662|CASA1_BOVIN</td><td>Alpha-S1-casein OS=Bos taurus GN=CSN1S1 PE=1 SV=2</td><td>99</td><td>YLGYLEQLLR</td><td></td><td></td><td>0,196</td><td>1266,893</td><td>634,454</td><td>1266,697</td><td>634,356</td><td>2</td><td>17</td><td>1.1.1.491.3</td><td>1</td><td>34,8903</td><td>0</td><td>-1</td></tr>
<tr><td>4</td><td>28,97</td><td>sp|P02662|CASA1_BOVIN</td><td>Alpha-S1-casein OS=Bos taurus GN=CSN1S1 PE=1 SV=2</td><td>35,21</td><td>HIQKEDVPSER</td><td>Propionamide@N-term</td><td>missed K-E@4</td><td>-0,580</td><td>1407,131</td><td>470,051</td><td>1407,711</td><td>470,244</td><td>3</td><td>9</td><td>1.1.1.346.4</td><td>1</td><td>21,5985</td><td>0</td><td>-1</td></tr>
<tr><td>4</td><td>28,97</td><td>sp|P02662|CASA1_BOVIN</td><td>Alpha-S1-casein OS=Bos taurus GN=CSN1S1 PE=1 SV=2</td><td>99</td><td>FFVAPFPEVFGK</td><td></td><td></td><td>0,180</td><td>1383,903</td><td>692,959</td><td>1383,723</td><td>692,869</td><td>2</td><td>13</td><td>1.1.1.496.3</td><td>1</td><td>35,4041</td><td>0</td><td>-1</td></tr>
<tr><td>4</td><td>28,97</td><td>sp|P02662|CASA1_BOVIN</td><td>Alpha-S1-casein OS=Bos taurus GN=CSN1S1 PE=1 SV=2</td><td>99</td><td>FFVAPFPEVFGK</td><td></td><td></td><td>0,192</td><td>1383,915</td><td>692,965</td><td>1383,723</td><td>692,869</td><td>2</td><td>12</td><td>1.1.1.497.3</td><td>1</td><td>35,5069</td><td>0</td><td>-1</td></tr>
<tr><td>4</td><td>28,97</td><td>sp|P02662|CASA1_BOVIN</td><td>Alpha-S1-casein OS=Bos taurus GN=CSN1S1 PE=1 SV=2</td><td>99</td><td>FFVAPFPEVFGK</td><td></td><td></td><td>0,170</td><td>1383,893</td><td>692,954</td><td>1383,723</td><td>692,869</td><td>2</td><td>11</td><td>1.1.1.498.3</td><td>1</td><td>35,6096</td><td>0</td><td>-1</td></tr>
<tr><td>4</td><td>28,97</td><td>sp|P02662|CASA1_BOVIN</td><td>Alpha-S1-casein OS=Bos taurus GN=CSN1S1 PE=1 SV=2</td><td>99</td><td>YLGYLEQLLR</td><td></td><td></td><td>0,240</td><td>1266,938</td><td>634,476</td><td>1266,697</td><td>634,356</td><td>2</td><td>12</td><td>1.1.1.493.3</td><td>1</td><td>35,0958</td><td>0</td><td>-1</td></tr>
<tr><td>4</td><td>28,97</td><td>sp|P02662|CASA1_BOVIN</td><td>Alpha-S1-casein OS=Bos taurus GN=CSN1S1 PE=1 SV=2</td><td>99</td><td>YLGYLEQLLR</td><td></td><td></td><td>-0,210</td><td>1266,487</td><td>634,251</td><td>1266,697</td><td>634,356</td><td>2</td><td>13</td><td>1.1.1.486.4</td><td>1</td><td>34,5504</td><td>0</td><td>-1</td></tr>
<tr><td>4</td><td>28,97</td><td>sp|P02662|CASA1_BOVIN</td><td>Alpha-S1-casein OS=Bos taurus GN=CSN1S1 PE=1 SV=2</td><td>99</td><td>YLGYLEQLLR</td><td></td><td></td><td>0,239</td><td>1266,937</td><td>634,476</td><td>1266,697</td><td>634,356</td><td>2</td><td>12</td><td>1.1.1.492.3</td><td>1</td><td>34,9931</td><td>0</td><td>-1</td></tr>
<tr><td>4</td><td>28,97</td><td>sp|P02662|CASA1_BOVIN</td><td>Alpha-S1-casein OS=Bos taurus GN=CSN1S1 PE=1 SV=2</td><td>99</td><td>YLGYLEQLLR</td><td></td><td></td><td>0,170</td><td>1266,867</td><td>634,441</td><td>1266,697</td><td>634,356</td><td>2</td><td>15</td><td>1.1.1.490.3</td><td>1</td><td>34,7876</td><td>0</td><td>-1</td></tr>
<tr><td>4</td><td>28,97</td><td>sp|P02662|CASA1_BOVIN</td><td>Alpha-S1-casein OS=Bos taurus GN=CSN1S1 PE=1 SV=2</td><td>99</td><td>YLGYLEQLLR</td><td></td><td></td><td>0,188</td><td>1266,886</td><td>634,450</td><td>1266,697</td><td>634,356</td><td>2</td><td>14</td><td>1.1.1.487.3</td><td>1</td><td>34,6184</td><td>0</td><td>-1</td></tr>
<tr><td>4</td><td>28,97</td><td>sp|P02662|CASA1_BOVIN</td><td>Alpha-S1-casein OS=Bos taurus GN=CSN1S1 PE=1 SV=2</td><td>93,01</td><td>YLGYLEQLLR</td><td></td><td></td><td>0,216</td><td>1266,913</td><td>634,464</td><td>1266,697</td><td>634,356</td><td>2</td><td>10</td><td>1.1.1.494.3</td><td>1</td><td>35,1986</td><td>0</td><td>-1</td></tr>
<tr><td></td><td></td><td></td><td></td><td></td><td></td><td></td><td></td><td></td><td></td><td></td><td></td><td></td><td></td><td></td><td></td><td></td><td></td><td></td><td></td></tr>
<tr><td>5</td><td>8,537</td><td>sp|P00760|TRY1_BOVIN</td><td>Cationic trypsin OS=Bos taurus PE=1 SV=3</td><td>99</td><td>SSGTSYPDVLK</td><td></td><td></td><td>-0,152</td><td>1152,414</td><td>577,215</td><td>1152,566</td><td>577,290</td><td>2</td><td>14</td><td>1.1.1.406.4</td><td>1</td><td>26,8717</td><td>0</td><td>-1</td></tr>
<tr><td>5</td><td>8,537</td><td>sp|P00760|TRY1_BOVIN</td><td>Cationic trypsin OS=Bos taurus PE=1 SV=3</td><td>99</td><td>SSGTSYPDVLK</td><td></td><td></td><td>-0,207</td><td>1152,359</td><td>577,187</td><td>1152,566</td><td>577,290</td><td>2</td><td>11</td><td>1.1.1.405.4</td><td>1</td><td>26,769</td><td>0</td><td>-1</td></tr>
</table>

Oxidation of hemoglobin with hydrogen peroxide (Perox)

N	%Cov	Accessions	Names	Conf	Sequence	Modifications	Cleavages	dMass	Prec MW	Prec m/z	Theor MW	Theor m/z	Theor z	Sc	Spectrum	Specific	Time	PrecursorSignal	PrecursorElution
1	32,65	sp|P02042|HBD_HUMAN	Hemoglobin subunit beta OS=Homo sapiens GN=HBB PE=1 SV=2	99	LLVVYPWTQR			0,318	1274,037	638,026	1273,718	637,866	2	10	1.1.1.696.3	1	50,0451	0	-1
1	32,65	sp|P02042|HBD_HUMAN	Hemoglobin subunit beta OS=Homo sapiens GN=HBB PE=1 SV=2	98,99	VLGAFSDGLAHLDNLK			0,072	1668,956	557,326	1668,884	557,302	3	11	1.1.1.689.4	1	49,3602	0	-1
1	32,65	sp|P02042|HBD_HUMAN	Hemoglobin subunit beta OS=Homo sapiens GN=HBB PE=1 SV=2	96,09	VVAGVANALAHKYH		missed K-Y@12	0,098	1448,887	483,970	1448,789	483,937	3	10	1.1.1.618.4	1	42,4696	0	-1
1	32,65	sp|P02042|HBD_HUMAN	Hemoglobin subunit beta OS=Homo sapiens GN=HBB PE=1 SV=2	95,14	LLVVYPWTQR			0,406	1274,124	638,069	1273,718	637,866	2	8	1.1.1.697.3	1	50,1524	0	-1
1	32,65	sp|P02042|HBD_HUMAN	Hemoglobin subunit beta OS=Homo sapiens GN=HBB PE=1 SV=2	78,05	LLVVYPWTQR			0,361	1274,079	638,047	1273,718	637,866	2	7	1.1.1.699.3	1	50,3232	0	-1
1	32,65	sp|P02042|HBD_HUMAN	Hemoglobin subunit beta OS=Homo sapiens GN=HBB PE=1 SV=2	76,79	LLVVYPWTQR			0,417	1274,135	638,075	1273,718	637,866	2	8	1.1.1.695.3	1	49,9422	0	-1
1	32,65	sp|P02042|HBD_HUMAN	Hemoglobin subunit beta OS=Homo sapiens GN=HBB PE=1 SV=2	70,9	LLVVYPWTQR			0,404	1274,122	638,068	1273,718	637,866	2	7	1.1.1.698.3	1	50,2552	0	-1
1	32,65	sp|P02042|HBD_HUMAN	Hemoglobin subunit beta OS=Homo sapiens GN=HBB PE=1 SV=2	95,92	VLGAFSDGLAHLDNLK			0,152	1669,036	557,353	1668,884	557,302	3	10	1.1.1.692.4	1	49,6687	0	-1
1	32,65	sp|P02042|HBD_HUMAN	Hemoglobin subunit beta OS=Homo sapiens GN=HBB PE=1 SV=2	94,67	VLGAFSDGLAHLDNLK	Deamidated(N)@14		-0,282	1669,585	557,536	1669,868	557,630	3	10	1.1.1.687.3	1	49,1197	0	-1
1	32,65	sp|P02042|HBD_HUMAN	Hemoglobin subunit beta OS=Homo sapiens GN=HBB PE=1 SV=2	31,81	VLGAFSDGLAHLDNLK	Deamidated(N)@14		-0,369	1669,499	557,507	1669,868	557,630	3	9	1.1.1.693.3	1	49,7365	0	-1
1	32,65	sp|P02042|HBD_HUMAN	Hemoglobin subunit beta OS=Homo sapiens GN=HBB PE=1 SV=2	28,67	VVAGVANALAHKYH		missed K-Y@12	0,151	1448,939	483,987	1448,789	483,937	3	7	1.1.1.617.4	1	42,3666	0	-1
2	31,91	sp|P69905|HBA_HUMAN	Hemoglobin subunit alpha OS=Homo sapiens GN=HBA1 PE=1 SV=2	99	FLASVSTVLTSK			0,092	1251,799	626,907	1251,707	626,861	2	9	1.1.1.664.4	0	46,8921	0	-1
2	31,91	sp|P69905|HBA_HUMAN	Hemoglobin subunit alpha OS=Homo sapiens GN=HBA1 PE=1 SV=2	99	IGGHGAEYGAEALER			0,037	1528,764	510,595	1528,727	510,583	3	14	1.1.1.596.4	0	40,4126	0	-1
2	31,91	sp|P69905|HBA_HUMAN	Hemoglobin subunit alpha OS=Homo sapiens GN=HBA1 PE=1 SV=2	90,03	MFLSFPTTK	Oxidation(M)@1		0,374	1086,916	544,465	1086,542	544,278	2	7	1.1.1.662.3	0	46,6514	0	-1
2	31,91	sp|P69905|HBA_HUMAN	Hemoglobin subunit alpha OS=Homo sapiens GN=HBA1 PE=1 SV=2	99	FLASVSTVLTSK			0,103	1251,811	626,913	1251,707	626,861	2	10	1.1.1.665.4	0	46,995	0	-1
2	31,91	sp|P69905|HBA_HUMAN	Hemoglobin subunit alpha OS=Homo sapiens GN=HBA1 PE=1 SV=2	40,76	MFLSFPTTK	Oxidation(M)@1		0,353	1086,895	544,455	1086,542	544,278	2	7	1.1.1.664.3	0	46,8573	0	-1
2	31,91	sp|P69905|HBA_HUMAN	Hemoglobin subunit alpha OS=Homo sapiens GN=HBA1 PE=1 SV=2	16,4	MFLSFPTTK	Oxidation(M)@1		0,055	1086,597	544,306	1086,542	544,278	2	6	1.1.1.661.4	0	46,5833	0	-1
